# Investigations of the Cobalt Hexamine Uranyl Carbonate System: Understanding the Influence of Charge and Hydrogen Bonding on the Modification of Vibrational Modes in Uranyl Compounds

**DOI:** 10.1021/acs.inorgchem.2c01982

**Published:** 2022-09-13

**Authors:** Mikaela Mary F. Pyrch, Jennifer L. Bjorklund, James M. Williams, Maguire Kasperski, Sara E. Mason, Tori Z. Forbes

**Affiliations:** Department of Chemistry, University of Iowa, Iowa City, Iowa 52242, United States

## Abstract

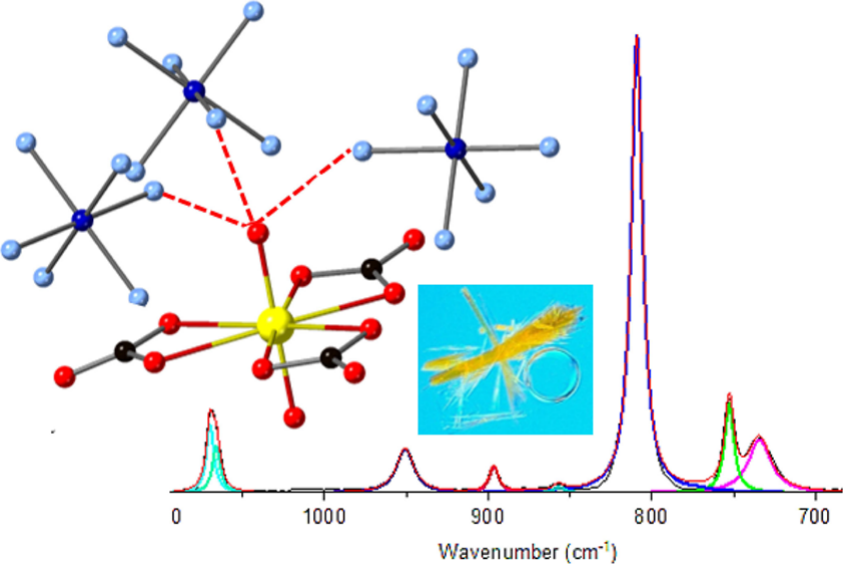

Hydrogen bonding networks within hexavalent uranium materials are complex and may influence the overall physical and chemical properties of the system. This is particularly true if hydrogen bonding takes places between the donor and the oxo group associated with the uranyl cation (UO_2_^2+^). In the current study, we evaluate the impact of charge-assisted hydrogen bonding on the vibrational modes of the uranyl cation using uranyl tricarbonate [UO_2_(CO_3_)_3_]^4–^ interactions with [Co(NH_3_)_6_]^3+^ as the model system. Herein, we report the synthesis and structural characterization of five novel compounds, [Co(NH_3_)_6_]Cl(CO_3_) (**Co_Cl_CO_3_**), [Co(NH_3_)_6_]_4_[UO_2_(CO_3_)_3_]_3_(H_2_O)_11.67_ (**Co4U3**), [Co(NH_3_)_6_]_3_[UO_2_(CO_3_)_3_]_2_Cl (H_2_O)_7.5_ (**Co3U2_Cl**), [Co(NH_3_)_6_]_2_[UO_2_(CO_3_)_3_]Cl_2_ (**Co2U_Cl**), and [Co(NH_3_)_6_]_2_[UO_2_(CO_3_)_3_]CO_3_ (**Co2U_CO_3_**), which contain differences in the crystalline packing and extended hydrogen bonding networks. We show that these slight changes in the supramolecular assembly and hydrogen bonding networks result in the modification of modes as observed by infrared and Raman spectroscopy. We use density functional theory calculations to assign the vibrational modes and provide an understanding about how uranyl bond perturbation and changes in hydrogen bonding interactions can impact the resulting spectroscopic signals.

## Abstract 2

In the current study, we evaluate the impact of charge-assisted hydrogen bonding on the vibrational modes of the uranyl cation using uranyl tricarbonate [UO_2_(CO_3_)_3_]^4−^ interactions with [Co(NH_3_)_6_]^3+^ as the model system.

## Introduction

Hydrogen bonding represents an important interaction in chemical systems, and the formation of hydrogen bond networks can directly influence chemical and physical properties of solid-state materials.^1−6,5−19^ The extent to which hydrogen bonding impacts the properties of high-valent actinide materials is of interest because of the unique nature of bonding within these complexes.^7^ Uranium is one of the most naturally abundant actinide elements and is commonly found in the hexavalent oxidation state in aqueous solutions and oxidizing conditions.^8^ Typically, U(VI) engages in covalent interactions with two oxygen atoms to create the linear triatomic uranyl cation [O=U(VI)=O]^2+^ that further coordinates to four, five, or six equatorial ligands to create a square, pentagonal, or hexagonal coordination geometry.^9,10^ Given the strong bonding within the actinyl unit, the *trans*-oxo groups are considered weak Lewis bases that do not readily engage in intermolecular interactions, including hydrogen bonding.^11^ For example, Watson and Hay utilized density functional theory (DFT) calculations to evaluate the geometries and energetics of the uranyl oxo group as a hydrogen bond acceptor and found that traditional hydrogen bond donors are actually repelled by the oxo groups in [UO_2_(H_2_O)_5_]^2+^.^12^

In a review compiled by Fortier and Hayton, instances where uranyl oxo groups interact with Lewis acids, including hydrogen atoms, were highlighted, yet this interaction does not seem to disrupt the uranyl bond to any extent as evidenced by U=O bond distances.^13^ This suggests that the intermolecular forces are quite weak and do not activate the oxo in any significant way. However, there are instances, such as in Pacman pyrrole-imine macrocycles, where the uranyl bond is perturbed by the presence of a hydrogen bond because of the specific ligand architecture.^14−16^ In addition, Watson and Hay observed that the identity of the equatorial ligand seems to play a role as the repellent nature of the uranyl oxo within [UO_2_(H_2_O)_5_]^2+^ can become attractive with the addition of nitrate groups to form [UO_2_(NO_3_)_2_(H_2_O)_2_]^0^.^12^ Therefore, it is important to consider what factors can increase the hydrogen bonding interaction to the uranyl oxo and how this impacts the observable properties of the material.

**Figure 1:**
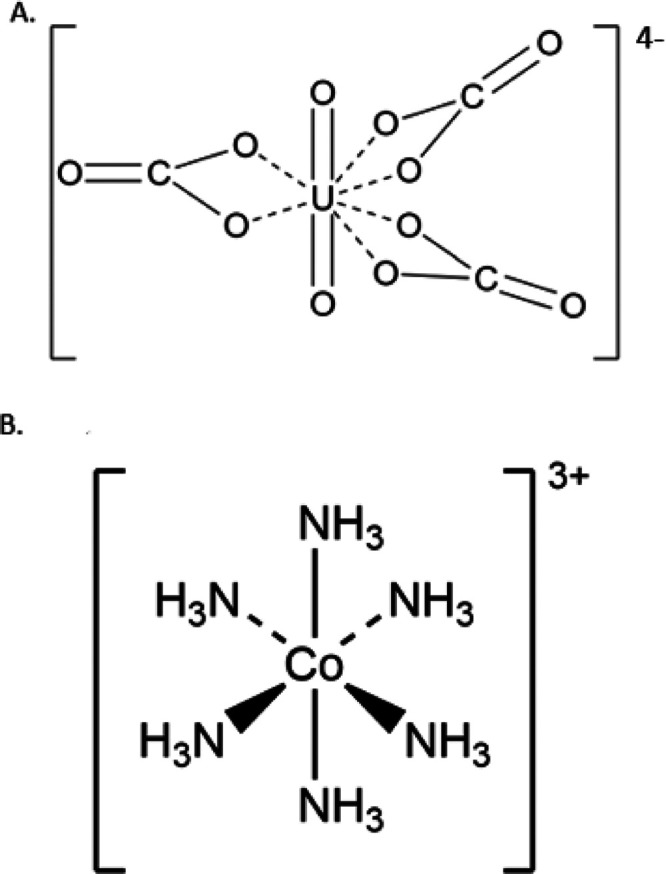
Molecular units of (A) uranyl tricarbonate and (B) cobalt hexamine that are utilized in this study to evaluate the impacts of charge-assisted hydrogen bonding on the U=O bond.

In the current study, we explore the influence of charge-assisted hydrogen bonding on the uranyl oxo by investigating uranyl tricarbonate coordination complexes [UO_2_(CO_3_)_3_]^4–^ crystallized with [Co(NH_3_)_6_]^3+^ (Figure 1). We hypothesized that a stronger hydrogen bond network, specifically charge-assisted hydrogen bonds, would more readily interact with the oxo atom, impact the overall bond strength within the uranyl cation, and influence the related vibrational spectroscopy of the solid-state material. Charge-assisted hydrogen bonds are unique because of the ionic character of the acceptor and donor atoms, which strengthen the electrostatic interaction of hydrogen bonds and have the potential to weaken and elongate the uranyl bond.^17^ Uranyl coordination compounds also have characteristic and identifiable vibrational spectra, where the symmetric (v_1_) and the asymmetric (v_3_) stretching bands of the uranyl are Raman- and IR-active, respectively. Positional changes or shifts in vibrational signals for characteristic UO_2_^2+^ bands are commonly attributed to the identity of the equatorial ligands, but additional activation of bands may occur in the presence of hydrogen bond networks.^18−20^ We hypothesized that the hydrogen bonding networks would lead to distortion of the uranyl bond and result in modification of the asymmetric and symmetric stretching band within the vibrational spectra. Uranyl carbonate compounds are good model systems because they have been studied thoroughly experimentally and computationally because of their importance in geologic environments, aqueous systems, and the nuclear fuel cycle.^8,21^ Cobalt hexamine represents an excellent hydrogen donor group because it possesses a high charge density at the metal center, and multiple hydrogen atoms are available for bonding interactions. Cobalt(III) hexamine has also been shown to engage in charge-assisted hydrogen bonding within biological systems and crystalline materials, including U(VI) compounds.^22−24^ In the current study, we report the structural characterization of five novel compounds with varied hydrogen bonding networks, [Co(NH_3_)_6_]Cl(CO_3_) (**Co_Cl_CO_3_**), [Co(NH_3_)_6_]_4_[UO_2_(CO_3_)_3_]_3_(H_2_O)_11.67_ (**Co4U3**), [Co(NH_3_)_6_]_3_[UO_2_(CO_3_)_3_]_2_Cl(H_2_O)_7.5_ (**Co3U2_Cl**), [Co(NH_3_)_6_]_2_[UO_2_(CO_3_)_3_]Cl_2_ (**Co2U_Cl**), and [Co(NH_3_)_6_]_2_[UO_2_(CO_3_)_3_]CO_3_ (**Co2U_CO_3_**). We utilize solid-state Raman and IR spectroscopy to then evaluate the influence of the structurally characterized hydrogen bonding network on the uranyl bond. In addition, we employ DFT calculations to perform geometric and vibrational analysis and calculate force constants which provide further insights into the impact of charge-assisted hydrogen bonding in solid-state U(VI) compounds.

## Experimental Methods

### Synthesis of Materials

Uranyl nitrate hexahydrate (UO_2_(NO_3_)_2_·6H_2_O) was purchased from Flinn Scientific Inc. **Caution!** Uranyl nitrate hexahydrate [UO_2_(NO_3_)_2_]·6H_2_O contains U-238, a naturally radioactive element. All uranium-bearing materials should be handled with standard precautions and by trained personnel. Tetramethyl ammonium hydroxide (TMAOH) 25% in water, cobalt(III) hexamine chloride ([Co(NH_3_)_6_]Cl_3_), and potassium carbonate (K_2_CO_3_) and sodium carbonate (Na_2_CO_3_) were purchased from Acros Organics, TCI, and Sigma-Aldrich, respectively. All chemicals were used as received, and stock solutions were prepared with millipure (18 MΩ) water.

#### [Co(NH_3_)_6_]Cl(CO_3_) (**Co_Cl_CO_3_**)

Equivalent amounts (1.0 mL) of a 0.18 M [Co(NH_3_)_6_]Cl_3_ solution were added to 0.20 M K_2_CO_3_ and millipure water in a 20 mL glass scintillation vial. The vial was left uncapped to encourage slow evaporation and reddish-brown crystals with a prismatic morphology formed over 12 h with a 60% yield based upon Co.

#### [Co(NH_3_)_6_]_4_[UO_2_(CO_3_)_3_]_3_H_2_O_11.67_ (**Co4U3**)

In a 20 mL glass scintillation vial, 0.18 M [Co(NH_3_)_6_]Cl_3_ (0.5 mL) was added to 1 mL of 0.2 M UO_3_ dissolved within the K_2_CO_3_ stock solution and 1 mL of millipure water. The solution slowly evaporated at room temperature, and orange blocks formed after 1 day. Percent yield of the **Co4U3** synthesis is 60% based upon Co.

#### [Co(NH_3_)_6_]_3_[UO_2_(CO_3_)_3_]_2_Cl H_2_O_7.5_ (**Co3U2_Cl**)

Aliquots of a 0.1 M uranyl nitrate stock solution (0.50 mL), 0.1 M TMAOH (0.15 mL), 0.46 M [Co(NH_3_)_6_]Cl_3_ (0.45 mL), and 1.0 mL of 1.0 M K_2_CO_3_ were combined in a 10 mL glass vial and placed in a refrigerator uncapped for 4 days. Orange crystals formed with a plate morphology with yields of <10% based upon Co.

#### [Co(NH_3_)_6_]_2_[UO_2_(CO_3_)_3_]Cl_2_ (**Co2U1_Cl**)

A 0.18 M [Co(NH_3_)_6_]Cl_3_ solution (1 mL) was combined with 1 mL of 0.2 M UO_3_ in 0.2 M K_2_CO_3_ and 3 mL of millipure water. The vial was left uncapped, and light orange columnar crystals formed after 1 day in yields of 48% based upon Co.

#### [Co(NH_3_)_6_]_2_[UO_2_(CO_3_)_3_]CO_3_H_2_O_3_ (**Co2U1_CO_3_**)

Aliquots of a 0.1 M uranyl nitrate stock solution (1 mL), 0.1 M TMAOH (0.3 mL), 0.18 M [Co(NH_3_)_6_]Cl_3_ (1 mL), and 1 mL of 1.0 M K_2_CO_3_ were combined in a 10 mL glass vial and left uncapped for 5 days. Orange crystals with a columnar morphology were produced in yields of 30% based upon Co.

Compounds **Co3U2_Cl** and **Co2U1_CO_3_** could also be synthesized without the addition of the carbonate anion if the solutions were left exposed to standard atmospheric conditions in the laboratory. For **Co3U2_Cl**, aliquots of the 0.1 M uranyl nitrate stock solution (1 mL), 0.1 M TMAOH (0.3 mL), and 0.01 M [Co(NH_3_)_6_]Cl_3_ (1 mL) were combined in a 20 mL glass scintillation vial. The pH of the solution was initially 12, and a solid yellow precipitate formed on the bottom of the vial. This initial solid dissolved after 18 h, and the clear orange solution was allowed to slowly evaporate for 3 days in an uncapped vial to produce orange plates of **Co3U2_Cl**. **Co2U1_CO_3_** formed from a similar synthetic condition where the 0.1 M uranyl nitrate stock solution (1.0 mL) and 0.1 M TMAOH (0.3 mL) were added to a 20 mL glass scintillation vial. In the case of **Co2U1_CO_3_**, a higher concentration of [Co(NH_3_)_6_]Cl_3_ (1.0 mL of 0.18 M) was also added to the solution. An initial yellow precipitate again formed and then re-dissolved after 24 h. After 3 days of slow evaporation in an uncapped vial, orange rods of **Co2U1_CO_3_** crystallized from the mother liquor.

This process fits well with what is currently understood for U(VI) chemistry under basic conditions (pH 12). Initially increasing the pH will result in U(VI) hydrolysis, which will cause precipitation of kinetically stable oxyhydroxide phase values.^25^ When water is in equilibrium with the atmosphere, it will contain dissolved CO_2_ at concentrations that are controlled by Henry’s law. This means that at pH 12, with 400 ppm CO_2_ in the air, we will reach levels greater than 10^–1^ moles of dissolved CO_2_ per liter (where dissolved CO_2_ is equal to H_2_CO_3_ + HCO_3_^–^ + CO_3_^2–^).^26^ At these concentrations, the uranyl tricarbonate phase is the only species present for U(VI) at these pH values.^25^

### Single-Crystal X-ray Diffraction

Single crystals of each coordination compound were visually identified on a polarized microscope, harvested from their respective mother liquors, and mounted on a MiTeGen MicroMount using NVH immersion oil (Cargille Labs). Structural information was collected on a Bruker D8 Quest single-crystal X-ray diffractometer equipped with a microfocus beam (Mo Kα; λ = 0.71073 Å) and an Oxford Systems low temperature cryosystem. Data were collected with the Bruker APEX3 software package,^27^ and peak intensities were corrected for Lorentz, polarization, background effects, and absorption. The structure solution was determined by intrinsic phasing methods and refined on the basis of *F*^2^ for all unique data using the SHELXTL version 5 series of programs.^28^ Metal atoms (U, Co) were located by direct methods, and the C, O, N, and Cl atoms were identified and modeled from the difference Fourier maps after partial refinement.

Many of the compounds contained positional disorder, which was accounted for by considering partial occupancy and split sites. **Co4U3** displayed disorder associated with the [UO_2_(CO_3_)]^4–^ complex that resulted in unreasonable U–U distances if the complex was fully occupied. Additional unit cell parameters were evaluated using CELLNOW, and doubling of the axes or lowering the symmetry of the space group did not rectify the positional disorder. The complex was successfully modeled using partial occupancy (50%) as evidenced by reasonable displacement parameters and bond distances/angles. **Co3U2_Cl** was originally solved in the hexagonal, *P*-3 space group, but significant disorder and unreasonable displacement parameters suggested that a lower symmetry space group was more appropriate. The lowest *R*_int_ value and most reasonable displacement values were achieved in an orthorhombic space group (*Cmma*) and resulted in the most agreeable thermal displacement parameters. **Co2U1_Cl** was placed in a triclinic *P*-1 space group, after multiple attempts to model the lattice Cl over several crystallographic positions did not provide proper thermal parameters or charge neutrality. **Co2U1_CO_3_** solved in the hexagonal space group *P*-3 and displayed disorder associated with one cobalt hexamine cation. One disordered water was also modeled as partially occupied over three positions in the lattice, while the interstitial carbonate anion required a DFIX constraint to enable reasonable C–O bond distances. Carbonate anions coordinated to the U(VI) atom in **Co2U1_CO_3_** also contained displayed disorder for one of the O atoms and were modeled as 50% occupied over two crystallographic positions.

Hydrogen atoms were included on all well-ordered NH_3_ and H_2_O molecules for the CoU compounds. A riding model was used to place the hydrogen atoms on the amine groups of the [Co(NH_3_)_6_]^3+^ cation, except for the Co(2) atom in **Co2U1_CO_3_** because there was significant disorder of the amine groups about the metal center. Water molecules within the lattice were also modeled with H atoms when possible, and the crystallographic positions of these atoms were in the difference Fourier map after modeling electron density of all the heavier atoms in the lattice. The bond distances and angles of the H atoms associated with the water molecules were restrained using DFIX and DANG commands.

Selected crystallographic parameters can be found in Table 1, and additional bonding information regarding the Co and interstitial anions/molecules can be found in the Supporting Information in Tables S1–S5. Images depicting the asymmetric unit with thermal ellipsoids for each of the compounds can also be found in the Supporting Information (Figures S1–S5). Crystallographic information files can be found on the Cambridge Structural Database by requesting numbers 2177491–2177495.

**Table 1:** Select Crystallographic Parameters for [Co(NH_3_)_6_]Cl(CO_3_) (**Co_Cl_CO_3_**), [Co(NH_3_)_6_]_4_[UO_2_(CO_3_)_3_]_3_(H_2_O)_11.67_ (**Co4U3**), [Co(NH_3_)_6_]_3_[UO_2_(CO_3_)_3_]_2_Cl (H_2_O)_7.5_ (**Co3U2_Cl**), [Co(NH_3_)_6_]_2_[UO_2_(CO_3_)_3_]Cl_2_ (**Co2U1_Cl**), and [Co(NH_3_)_6_]_2_[UO_2_(CO_3_)_3_]CO_3_(H_2_O)_3_ (**Co2U1_CO_3_**)

	Co_Cl_CO_3_	Co4U3	Co3U2_Cl	Co2U1_Cl	Co2U1_CO_3_
empirical formula	CN_6_H_18_O_3_CoCl	C_9_N_24_H_69_O_44.67_U_3_Co_4_	C_6_N_18_H_69_O_29.5_U_2_Co_3_Cl	C_3_N_12_H_36_O_14_UCo_2_Cl_2_	C_4_N_12_H_42_O_17_Uco_2_
formula weight	256.56	2178.14	1553.73	891.13	886.16
space group	*P*2_1_3	*P*2_1_/*n*	*Cmma*	*P*-1	*P*-3
*a* (Å)	9.9014(5)	16.978(5)	25.2392(19)	6.8093(3)	15.5979(5)
*b* (Å)	9.9014(5)	7.780(3)	15.1709(13)	12.5621(7)	15.5979(5)
*c* (Å)	9.9014(5)	23.796(7)	12.9936(13)	14.2362(7)	6.5340(2)
α (°)	90	90	90	92.979(2)	90
β (°)	90	95.835	90	91.030(2)	90
γ (°)	90	90	90	103.331(2)	90
*V* (Å^3^)	970.7(1)	3127.0(2)	4975.3(8)	1182.77(10)	1376.7(1)
Z	4	2	4	2	1
ρ (g/cm^3^)	1.756	8.892	7.613	8.515	7.143
μ (mm^–1^)	2.030	2.334	1.982	2.413	2.102
*F*(000)	536	2103	2720	828	834
θ range (°)	2.909–25.983	2.412–26.143	2.250–26.369	2.136–26.110	3.464–26.839
limiting indices	–12 ≤ *h* ≤ 12	–21 ≤ *h* ≤ 21	–31 ≤ *h* ≤ 31	–8 ≤ *h* ≤ 8	–19 ≤ *h* ≤ 19
	–12 ≤ *k* ≤ 12	–9 ≤ *k* ≤ 9	–18 ≤ *k* ≤ 18	–15 ≤ *k* ≤ 15	–19 ≤ *k* ≤ 19
	–12 ≤ *l* ≤ 12	–29 ≤ *l* ≤ 29	–16 ≤ *l* ≤ 16	–17 ≤ *l* ≤ 17	–8 ≤ *l* ≤ 8
refl. collected/unique	34,609/637	87,272/6222	123,734/2707	32,224/4535	20,496/1969
*R*_int_	0.0521	0.0658	0.0513	0.0384	0.0568
data/restraints/parameters	650/6/39	6222/12/479	2707/148/0	4708/0/331	1969/3/122
GOF on *F*^2^	1.190	1.059	1.089	1.115	1.253
final *R* indices	*R*_1_ = 0.0182	*R*_1_ = 0.0227	*R*_1_ = 0.0543	*R*_1_ = 0.0242	*R*_1_ = 0.0345
[*I* > 2σ(*I*)]	*wR*_2_ = 0.0477	*wR*_2_ = 0.0555	*wR*_2_ = 0.1635	*wR*_2_ = 0.0579	*wR*_2_ = 0.1007
*R* indices (all data)	*R*_1_ = 0.0192	*R*_1_ = 0.0273	*R*_1_ = 0.0568	*R*_1_ = 0.0257	*R*_1_ = 0.0361
	*wR*_2_ = 0.0488	*wR*_2_ = 0.0576	*wR*_2_ = 0.1671	*wR*_2_ = 0.0579	*wR*_2_ = 0.1014
largest peak and hole	0.220 to −0.454	1.286 to −0.782	2.965 to −5.237	2.209 to −2.058	2.538 to −1.154

### Powder X-ray Diffraction

Purity of the bulk crystalline material was confirmed by powder X-ray diffraction using a Bruker D-5000 Advanced Powder Diffractometer equipped with Cu Kα radiation (λ = 1.5418 Å) and a LynxEye solid-state detector. Data were collected from 2 to 40° 2θ with a step size of 0.02° 2θ and a count time of 0.5 s/step. Predicted X-ray diffraction patterns were plotted using the Mercury Software version 3.1 and compared to the experimental data. Diffractograms of the experimental and calculated patterns can be found in the Supporting Information (Figures S6–S10).

### Vibrational Spectroscopy

Solid-state compounds were analyzed by Fourier transform infrared (FTIR) and Raman spectroscopy. After confirming the bulk purity of the material, approximately 5 mg of the sample was mixed with KBr and pressed into translucent disks for analysis on a Nicolet Nexus 760 FTIR Spectrometer. Infrared spectra were collected from 500 to 4000 cm^–1^ with a resolution of 2 cm^–1^. Solid-state Raman spectra were collected on a SnRI High-Resolution Sierra 2.0 Raman spectrometer equipped with 785 nm laser energy and 2048 pixels TE-cooled CCD. Laser power was set to the maximum output value of 15 mW, and the system was configured to acquire data by the Orbital Raster Scanning mode, giving the highest achievable spectral resolution of 2 cm^–1^. Each sample was irradiated for an integration time of 60 s and automatically reiterated six times in multiacquisition mode. The average of the six Raman spectra collected for a sample is reported as the final Raman spectrum. Because of smaller yields, the solid-state Raman spectra for **Co4U3** and **Co2U1** were collected on a Renishaw inVia confocal Raman microscope with a Leica DM2700 series microscope using a 785 nm laser and a CCD detector. Each sample was isolated, mounted to a glass slide using double-sided tape, and loaded onto the sample stage. Laser focusing was performed by utilizing the confocal microscope, and the spectra were collected from 200 to 3000 cm^–1^. To accurately process the vibrational spectra, the background was subtracted, multiple peaks were fit using the peak analysis protocol with Gaussian functions, and all the fitting parameters converged with a chi-squared tolerance value of 10^–14^ in the OriginPro 9.1.0 (OriginLab, Northampton, MA) 64-bit software.^29^

### DFT Methods

DFT calculations were used to gain a deeper understanding of the hydrogen bonding interactions that occur within these systems. Initial geometries for the DFT calculations were isolated molecular models generated based on the experimental crystal information files (CIFs) obtained from the structural analysis of the **CoU** compounds. Because of the disorder present in Co3U2_Cl, only the distances and geometries of central atoms were considered. This molecular approach allows us to systematically induce subtle structural changes in the coordination environment of the uranyl cation and incrementally increase the H-bonding present, which affords for a methodical analysis of the roles these features have on the vibrational spectroscopy. Full geometry optimization and vibrational analysis calculations were conducted using the Becke 3-parameter Lee-Yang-Par (B3-LYP) hybrid functional within the TURBOMOLE 7.2 software package and the default triple-zeta valence polarized (def-TZVP) basis set for U, Co, C, O, and N atoms.^30−33^ SCF energy converged to at least 0.3 meV, and forces were converged to a minimum of 5 meV Å^–1^. The potential between system electros and U is accounted for using the small-core (60 core electrons) relativistic effective core potential (RECP) by Dolg and co-workers.^34^ The isolated molecular models were embedded in the continuum solvent model COSMO with a dielectric constant (ε) of 78.54 to simulate aqueous solvent contributions to the electrostatics.^35^

To systematically explore how the ν_1_ and ν_3_ uranyl stretching modes are influenced by perturbations to the bonding environment, two DFT studies utilized fixed geometries paired with vibrational analysis. Both the free UO_2_^2+^ cation and then the [UO_2_(CO_3_)_3_]^4–^ complex were isolated from the **Uco** compounds and then allowed to relax to the energy minimized form. Then the U=O bonds in both complexes were varied in step sizes of 0.02 Å, and the vibrational analysis was performed to explore the impact on the position of the stretching modes. A second series of calculations evaluated the effects of counter-cation interactions on the U=O stretching bands by fixing the [Co(NH_3_)_6_]^3+^ positions relative to the [UO_2_(CO_3_)_3_]^4–^_._ Jaquet and Haeuseler reported similar methodologies as a means to evaluate simulate coordination environments that were associated with the crystallographic positions.^36^ Unconstrained [UO_2_(CO_3_)_3_]^4–^ + [Co(NH_3_)_6_]^3+^ calculations were first fully optimized, and subsequent molecular models fixed the positions of the [Co(NH_3_)_6_]^3+^ cations according to data obtained from the structural characterization of the solid UCo compounds. Vibrational modes were calculated in these specific environments to evaluate changes in the expected spectral features. For all calculations, SCF energy was converged to at least 0.3 meV, and forces were converged to at least 5 meV Å^–1^.

## Results and Discussion

### Structural Analysis

The first reported compound (**Co_Cl_CO_3_**) does not contain U(VI) within the crystalline lattice but serves as a model compound for the spectral signals associated with [Co(NH_3_)_6_]^3+^ and CO_3_^2–^ ions within the solid phase (Figure SI1). This cobalt hexamine complex contains six Co–N bonds at distances of 1.963(2) to 1.964(2) Å, and the crystalline lattice contains a single chloride anion and a carbonate anion with C–O bond distances of 1.287(2) Å. Hydrogen bonding occurs between the H atoms on the amine groups and oxygen atoms on the carbonate anion with donor to acceptor (D-H···A) distances ranging from 2.848 to 3.004 Å.

Each of the other four compounds reported in this study contains the [UO_2_(CO_3_)_3_]^4–^ coordination complex and exhibits subtle differences in U=O bond distances (Table 2). In all cases, the U(VI) cation is strongly bound to two oxygen atoms to create the nearly linear dioxo cation (UO_2_^2+^) with bond lengths ranging from 1.757(11) to 1.801(4) Å. Three of the four compounds contain symmetric U=O bond lengths within the uranyl moiety, and only in the case of **Co3U2_Cl** do we notice a slight asymmetry in the uranyl bonds, with a difference of 0.02 Å. Therefore, we did not observe significant asymmetry in the uranyl bond within any of the compounds presented herein. In all cases, three carbonate anions surround the uranyl cation through the equatorial plane in a bidentate coordination mode. Equatorial bond distances within this metal complex range from 2.393(4) to 2.461(6) Å among the four compounds. This leads to an overall hexagonal bipyramidal coordination geometry and results in the [UO_2_(CO_3_)_3_]^4–^ species.

**Table 2:** Summary of Bond Distances for the [UO_2_(CO_3_)_3_]^4–^ Complex in **Co4U3**, **Co3U2_Cl**, **Co2U1_Cl**, and **Co2U1_CO_3_** and Literature Values for Bond Distances and Reported Vibrational Bands for the Uranyl Cation within Coordination Compounds Containing [UO_2_(CO_3_)_3_]^4–^

compound	U=O axial (Å)	U–O equatorial (Å)	reported UO_2_^2+^ spectral modes (cm^–1^)	reference
**Co4U3**	1.796(3), 1.798(3)	2.412(3)–2.434(3)	805^a^	this work
	1.781(5), 1.793(5)	2.409(6)–2.461(6)		
**Co3U2_Cl**	1.76(1), 1.78(1)	2.415(9)–2.423(7)	809^a^	this work
**Co2U1_Cl**	1.798(4), 1.801(4)	2.393(4)- 2.438(4)	807^a^	this work
**Co2U1_CO_3_**	1.771(8), 1.776(8)	2.435(5)–2.439(5)	806^a^	this work
NH_4_[(UO_3_)(CO_3_)_3_]	1.79(1)	2.44(1)–2.46(1)	831, 883	Graziani et al.,^37^ Čejka, Novitskiy et al.^38,48^
[C(NH_2_)_3_]_4_[(UO_2_)(CO_3_)_3_]	1.78(1), 1.80(2)	2.440 (6)–2.451(2)	831(ν_1_), 892(ν_3_)	Fedoseev et al.,^58^ Allen et al.^39^
[N(CH_3_)_4_]_4_[(UO_3_)(CO_3_)_3_] (H_2_O)_8_	1.803(3), 1.814(3)	2.418(3)–2.450(3)		Reed et al.^40^
Na_4_[UO_2_(CO_3_)_3_]	1.807(5), 1.814(5)	2.385(4)–2.427(3)	810, 816(ν_1_), 843(ν_3_)	Li et al.,^79^ Čejka^38,41^
Na_2_Ca[(UO_2_)(CO_3_)_3_](H_2_O)_6_	1.81(2), 1.78(2)	2.41(1)–2.46(1)	833(ν_1_), 919 (ν_3_)	Coda et al.,^43^ Driscoll et al.^80^
Na_2_Rb_2_[UO_2_(CO_3_)_3_]	1.779(8)	2.418(8)–2.433(6)		Kubatko and Burns^44^
Na_6_Mg[UO_2_(CO_3_)_3_]_2_(H_2_O)_6_	1.792(6)	2.392(7)–2.486(7)		Olds et al.^45^
Mg_2_[(UO_2_)(CO_3_)_3_](H_2_O)_18_	1.788(4), 1.785(4)	2.419(4)–2.457(4)	822, 875	Mayer and Mereiter,^42^ Colmenero et al.,^46^ Amayri et al.^47^
K_4_[(UO_2_)(CO_3_)_3_]	1.802	2.425(4)–2.434 (4)	815, 881	Anderson et al.,^81^ Novitskiy et al.^48^
K_2_Ca_3_[(UO_2_)(CO_3_)_3_]_2_(H_2_O)_8_	1.781(4), 1.769(4)	2.423(4)–2.444(4)		Plášil et al.^49^
	1.791(4)	2.400(4)–2.454(4)		
	1.775(4), 1.798(4)	2.404(4)–2.441(4)		
Rb_4_[(UO_2_)(CO_3_)_3_]	1.79(1)	2.43(1)–2.45(1)	828, 877	Chernorukov et al.,^50^ Gorbenko-Germanov and Zenkova^51^
Cs_4_[(UO_2_)(CO_3_)_3_]	1.806(4)	2.420(4) -2.435(4)	808, 877	Krivovichev, Burns, Gorbenko-Germanov and Zenkova^51,52^
Ca_2_[(UO_2_)(CO_3_)_3_](H_2_O)_11_	1.784(7), 1.774(7)	2.417(6)–2.448(7)	822, 902, 885, 883	Mereiter^53^
Ca_9_[(UO_2_)(CO_3_)_3_]_4_(CO_3_)(H_2_O)_28_	1.773(9), 1.779(9)	2.411(5)–2.481(5)		Kampf et al.^54^
	1.76(9), 1.773(9)	2.416(6)–2.457(5)		
CaMg[(UO_2_)(CO_3_)_3_](H_2_O)_12_	1.777(3), 1.788(3)	2.412(3)–2.457(3)		Mereiter^55^

a See Table 3 for additional information on the uranyl stretching modes associated with these compounds.

Notable differences in the structural arrangement within the **CoU** compounds are variations in the molar ratio of the cobalt hexamine cation and the uranyl tricarbonate anion. In the case of **Co4U3**, we observe a Co:U ratio of 1.33 to give a formula of [Co(NH_3_)_6_]_4_[UO_2_(CO_3_)_3_]_3_, and no additional charge balancing anions or cations are necessary to neutralize the overall charge of the compound. Water molecules are located throughout the crystalline lattice of **Co4U3** that results in the overall structural formula of [Co(NH_3_)_6_]_4_[UO_2_(CO_3_)_3_]_3_H_2_O_11.67_. Increasing the Co:U ratio to 1.5 in **Co3U2_Cl** results in the need to include additional charge balancing anions within the lattice, and we determined that the structure contained an additional Cl^–^ anion located within four partially occupied sites. The Cl^–^ anion is present in significant quantities because of the addition of the cobalt hexamine chloride reagent. Additional water molecules are again present, and the overall formula for **Co3U2_Cl** is [Co(NH_3_)_6_]_3_[UO_2_(CO_3_)_3_]_2_Cl(H_2_O)_7.5_. Both **Co2U1_Cl** and **Co2U1_CO_3_** possessed a Co:U ratio of 2:1 and required an additional −2 charge compensation to create neutrality. The negative charge is achieved through the incorporation of either two Cl^–^ (**Co2U1_Cl)** or one CO_3_^2–^ anion (**Co2U1_CO_3_**) and results in overall formulas of [Co(NH_3_)_6_]_2_[UO_2_(CO_3_)_3_]Cl_2_ and [Co(NH_3_)_6_]_2_[UO_2_(CO_3_)_3_]CO_3_(H_2_O)_3_, respectively.

Comparisons between the **CoU** compounds and other uranyl tricarbonate phases reported in the literature indicated similarities in relative ratios of charge balancing constituents and bond distances (Table 2). The uranyl (U=O) bond lengths in this class of compounds ranged from 1.73(4) to 1.85(4) Å, and the U–O equatorial distances occurred between 2.38(4) to 2.48(4) Å. All **CoU** compounds exhibit bond distances within this range. A majority of the uranyl tricarbonate compounds exhibited symmetric U=O bond lengths within the uranyl moiety, except in the case of the mineral Paddlewheelite (MgCa_5_Cu_2_[(UO_2_)(CO_3_)_3_]_4_(H_2_O)_33_).^45^ In this case, there is significant asymmetry in the uranyl bond that ranges from 0.02 to 0.07 Å. The largest asymmetry in the U=O bond (0.07 Å) within Paddlewheelite occurs in the region where there are significant differences in the intermolecular interactions that occur between the oxo groups and neighboring cations and hydrogen bond donors. However, most of the previously reported uranyl bond asymmetry is similar to the value observed within the **Co3U2_Cl** coordination compound (0.02 Å).

**Figure 2:**
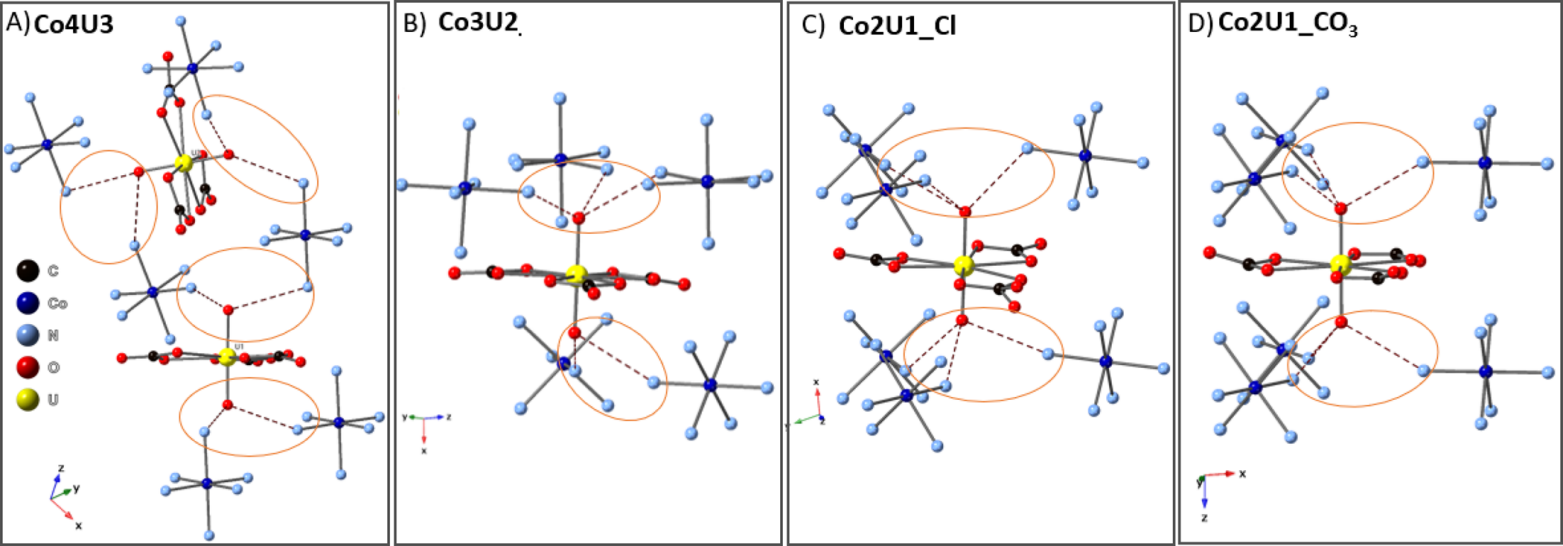
Hydrogen bonding for the cobalt hexamine to the uranyl tricarbonate compounds, showing differences in the arrangement of the [Co(NH_3_)_6_]^3+^ counterions and hydrogen bonding networks for **Co4U3**, **Co3U2_Cl**, **Co2U1_Cl**, and **Co2U1_CO3**. The U, Co, Cl, O, N, and C atoms are depicted as yellow, dark blue, green, red, light blue, and black spheres, respectively. The H atoms have been removed for clarity. Hydrogen bonding is illustrated using dashed red lines.

Intermolecular interactions within the **CoU** compounds occur through charge-assisted hydrogen bonding that takes place between the cobalt hexamine donors and the uranyl oxo acceptor groups. An extensive hydrogen bonding network is noted within the solid-state compounds, and significant differences are observed based upon the arrangement of the [Co(NH_3_)_6_]^3+^ cations (Figure 2). **Co4U3** displays symmetric bifurcated hydrogen bonding between the uranyl oxo groups and the [Co(NH_3_)_6_]^3+^ cations (Figure 2a). Hydrogen bonding distances are relatively long, with distances ranging from 2.96 to 3.01 Å. Stronger interactions occur between the carbonate anions and water molecules located in the interstitial region (2.734–2.809 Å). The uranyl oxo groups in compound **Co3U2_Cl** engage in asymmetric H-bonding interactions because of the arrangement of the counterions within the layers. We note that in this compound, the O1 atom acts as a H bond acceptor to two different donors (N6) at a donor to acceptor distance (D–H···A) of 2.96 Å (Figure 2b). Donor (N5) to acceptor (O2) distances for the hydrogen bonding interactions occurring at the second oxo group are similar in distance (2.94 Å), but again the uranyl bond distance is asymmetric. Hydrogen bonding in compound **Co2U1_CO_3_** follows the symmetric nature of the [Co(NH_3_)_6_]^3+^ cations and exhibits interactions to oxygen acceptors on the uranyl moiety and carbonate anion (Figure 2d). Each oxo group (O1 and O2) interacts in a symmetric fashion to hydrogen atoms on the cobalt hexamine cation with D–H···A distances of 2.97 and 3.01 Å, respectively. The arrangement of the [Co(NH_3_)_6_]^3+^ cations around the oxo groups leads to trifurcated H-bonding to each layer of [UO_2_(CO_3_)_3_]^4–^. In addition, the carbonate anions also participate in H-bonding with the oxygen atoms (O3, O4, and O5) linked to the U(VI) metal center. Again, the uranyl bond distance is symmetric, and each O atom can interact with H atoms located above and below the uranyl tricarbonate complex with D–H···A distances ranging from 2.95 to 3.00 Å. **Co2U1_Cl** (Figure 2c) shows a similar hydrogen bonding network to **Co2U1_CO_3_**, with slight differences in the donor to acceptor distances (2.893–3.28 Å).

Synthetic compounds containing the uranyl tricarbonate anion and hydrogen bond donors have been previously reported, but there is little evidence of this type of interaction occurring with the uranyl oxo groups in these materials. The ammonium cation can cocrystallize with the [UO_2_(CO_3_)_3_]^4–^ complex and engages in medium strength H-bonding based upon Jeffrey’s classification (D–H···A = 2.85–3.22 Å).^37,56^ All the NH_4_^+^ cations were located either below and above the equatorial plane of the uranyl cation but only participate in H-bonding with the O atoms of the bound CO_3_^–^ anions. A H-bonding network within the tetramethylammonium uranyl tricarbonate compound has also been delineated, and again the uranyl oxo groups do not participate in additional intermolecular interactions with the hydrogen bond donors.^57^ Within the guanidinium system, the trimeric species [(UO_2_)_3_(CO_3_)_6_]^6–^ was the major species isolated, but [C(NH_2_)_3_]_4_[(UO_2_)(CO_3_)_3_] has also been reported by Fedosseev and co-workers.^58^ In both cases, no hydrogen bonds were observed between the guanidinium cations and the uranyl oxo groups.

Evaluation of the uranyl carbonate literature suggests that the **CoU** compounds are unique in that the uranyl oxo groups do participate in the hydrogen bonding network created by the cobalt hexamine cation. As mentioned in the Introduction section, the cobalt hexamine cation was chosen specifically to engage the uranyl oxo groups because of the high charge density associated with the complex. We observe this to occur within the compounds presented herein, and the H-bonds can all be classified using the categories delineated by Jeffrey as medium strength.^59,60^ The [Co(NH_3_)_6_]^3+^ cation has also been previously reported to crystallize other uranyl coordination complexes, including substituted malonato and tetrahydroxide complexes.^61−63^ Extensive hydrogen bonding networks occur within both systems that include interactions between the amine and uranyl oxo groups; however, the hydrogen bonding strength is much weaker for the malonato and tetrahydroxide complexes (average D–H···A distances = 3.2(1) Å). Clark et al. discussed the H-bonding interactions in relation to the uranyl oxo distances, pointing out that the shortest bond (1.802(6) Å) showed only one interaction to the [Co(NH_3_)_6_]^3+^ unit and the longest U=O bond at 1.835(5) Å possessed multiple hydrogen bonds.^63^

Within the evaluation of [Co(NH_3_)_6_]_2_[UO_2_(OH)_4_)]_3_H_2_O, Clark et al. also noted that there was a 10 cm^–1^ difference between uranyl symmetric stretching (ν_1_) mode of the solid and that of the related solution phase. It was suggested that this difference could be due to variability in the number of hydro ligands attached the uranyl cation or the impact of hydrogen bonding within the solid-state material. Additional experimental and computational analysis has indicated that the uranyl tetrahydroxide is the dominant species under alkaline conditions, so the impact of hydrogen bonding is the likely explanation of this spectral variability.^64,65^ Thus, we turn to vibrational analysis to further identify the impact of hydrogen bonding within the **CoU** materials.

### Vibrational Spectroscopy

**Figure 3:**
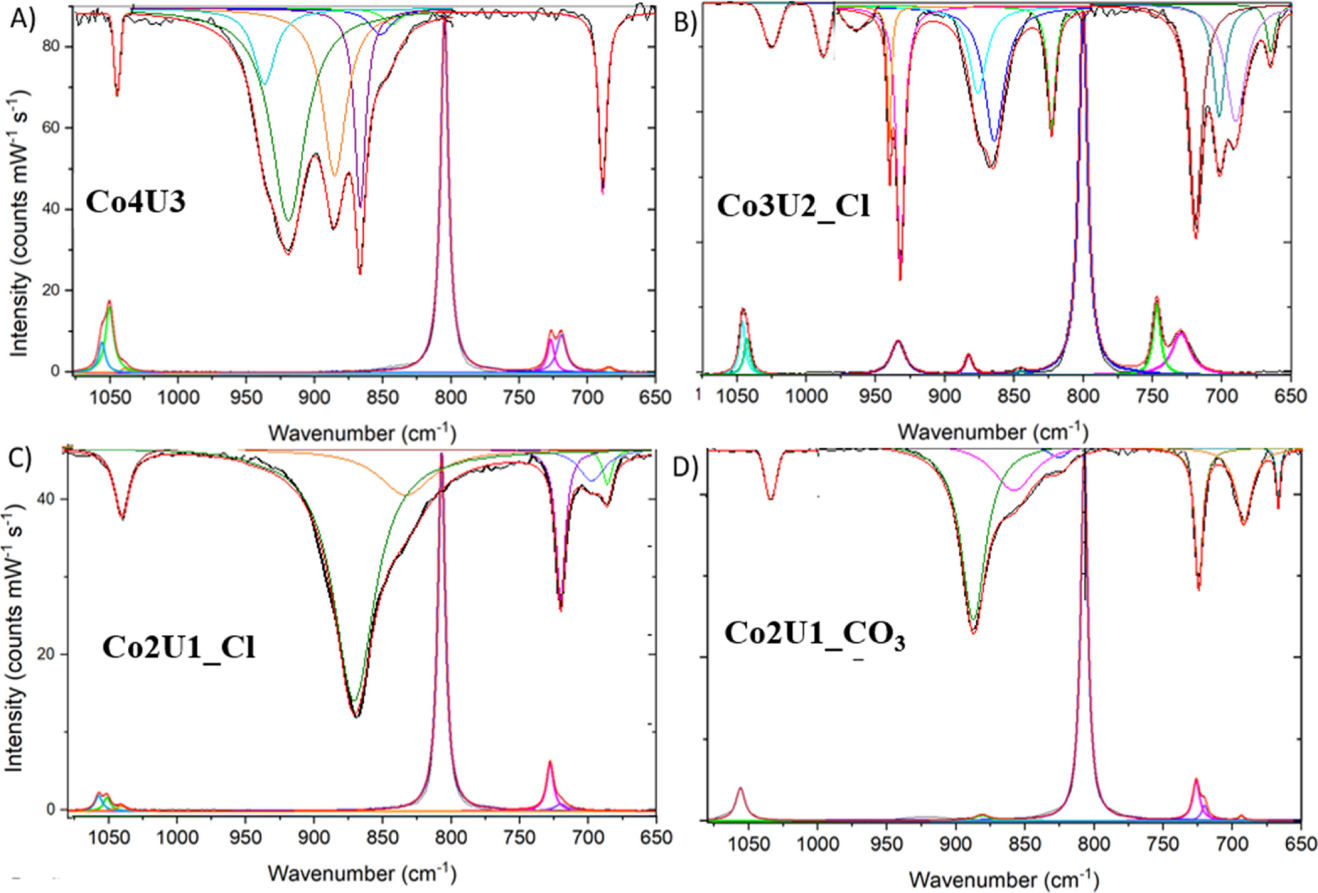
Solid-state vibrational spectra of (a) **Co4U3**, (b) **Co3U2_Cl**, (c) **Co_2_U1_Cl**, and (d) **Co2U1_CO_3_**. IR spectra are on top, while Raman spectra are located on the bottom for each sample.

For this work, we will focus specifically on the uranyl symmetric stretch (ν_1_) and asymmetric stretch (ν_3_) associated with the uranyl cation. If one considers the uranyl point group symmetry to be *D*_∞*h*_, then the symmetric and asymmetric stretches are predicted to be Raman- and IR-active, respectively. However, U=O bond perturbation can result in lower symmetry of the uranyl cation through either bond asymmetry (*C*_∞*v*_) or bending (*C*_2*v*_) that would result in activation of both the ν_1_ and ν_3_ bands in the Raman and IR spectra. Additional combination modes with the carbonate ligands and the cooperative nature of the hydrogen bonding network can also influence the spectral signals.^46^ To focus specifically on these issues, we evaluated the spectral window 700–1100 cm^–1^ to capture major features of the uranyl, carbonate, and cobalt hexamine components (Figure 3 and Table 3). Assignments were determined based upon the DFT spectral band analysis. Additional spectral features associated with the [Co(NH_3_)_6_]^3+^ are observed between 300 and 500 cm^–1^, and full spectra are provided in the Supporting Information (Figure S8).

**Table 3:** Observed Raman Frequencies for Solid-State Raman Spectra and the IR Frequencies of [Co(NH_3_)_6_]Cl(CO_3_) (**Co_Cl_CO_3_**), [Co(NH_3_)_6_]_4_[UO_2_(CO_3_)_3_]_3_(H_2_O)_11.67_ (**Co4U3**), [Co(NH_3_)_6_]_3_[UO_2_(CO_3_)_3_]_2_Cl(H_2_O)_7.5_ (**Co3U2_Cl**), Co(NH_3_)_6_]_2_[UO_2_(CO_3_)_3_]Cl_2_ (**Co2U1_Cl**), and [Co(NH_3_)_6_]_2_[UO_2_(CO_3_)_3_]CO_3_(H_2_O)_3_ (**Co2U1_CO_3_**) within the Spectral Window of Interest (500–1100 cm^–1^)

**Co_Cl_CO_3_**		**Co4U3**		**Co3U2_Cl**		**Co2U1_Cl**		**Co2U1_CO_3_**		assignment
R	IR	R	IR	R	IR	R	IR	R	IR	
				1070						NH_3_ breathing
1053		1058		1065		1057		1056		ν_4_CO_3_^2–^ breathing
		1052	1054		1049	1051				ν_4_CO_3_^2–^ breathing + NH_3_ twist
		1041			1008	1041	1044		1043	ν_4_CO_3_^2–^ breathing + NH_3_ twist
					957					ν_3_UO_2_^2+^ + NH_3_ twist
				950	948					ν_3_UO_2_^2+^ + NH_3_ twist
			940							NH_3_ twist + ν_3_UO_2_^2+^
			922							NH_3_ twist + ν_3_UO_2_^2+^
			888	896	889			881	888	NH_3_ twist + ν_3_UO_2_^2+^
			869		876		871			NH_3_ twist + ν_3_UO_2_^2+^
			854	856					859	NH_3_ rocking
	832		845		832		833			NH_3_ rocking
									827	CO_3_^2–^
		805		809		807		806		ν_1_UO_2_^2+^ + CO_3_ wag
				753						NH_3_ breathing + ν_1_UO_2_^2+^
		727		734		728		726		ν_1_UO_2_^2+^ + ν_2_CO_3_
		719			721	720	719	720	722	ν_2_CO_3_^2–^
					704					H_2_O libration
		684	690		692		696	693	691	ν_2_CO_3_^2–^ + NH_3_ twist
							685		668	H_2_O libration

Variability in the spectral signals is observed in the CoU compounds that are associated with differences in the hydrogen bonding network. **Co_Cl_CO_3_** only exhibits one band in the spectral window of interest (1053 cm^–1^) that corresponds to the ν_4_ CO_3_^2–^ breathing mode. In the presence of UO_2_^2+^, multiple bands are observed which correspond to concerted motion. Some of these bands (726–734 cm^–1^) are associated with concerted motions between the uranyl cation and the bound carbonate anion. In addition, the hydrogen bonding interactions between the uranyl oxo groups and the cobalt hexamine cation lead to a concerted uranyl stretching with NH_3_ twisting motions (753, ∼805, 856, 881, and 891 cm^–1^). Similar hydrogen bonding interactions the amine group and the carbonate anion also exist and lead to multiple bands associated with the CO_3_^2–^ breathing modes between 1041 and 1065 cm^–1^.

One notable band in **Co3U2_Cl** is located at 950 cm^–1^ and could be assigned to the activated ν_3_ asymmetric stretching vibration for the uranyl cation, twisting of the amine group associated with the [Co(NH_3_)_6_]^3+^ cation, or a combination of the two modes together. This modified vibrational signal does pair with the subtle asymmetry of the uranyl bond length noted in this compound that may lower the overall point group symmetry and lead to an observable peak in the Raman spectra. However, a difference of only 0.02 Å is quite small, and this asymmetry alone may not account for this specific band. Thus, it is more likely that it is associated with the concerted NH_3_ twisting and asymmetric stretching of the uranyl that gives rise to the band in the spectra.

Infrared spectroscopy was also performed for all compounds and allows us to confirm the position of the ν_3_ bands. Multiple bands are present in the 870–960 cm^–1^ region that correspond to concerted motions between the amine and the uranyl oxo groups. Both **Co2U1** compounds contain fewer bands in this region and may be related to the trifurcated, symmetric bonding that exists between the cobalt hexamine and uranyl oxo groups. **Co4U3** and **Co3U2_Cl** contain six and five modes within that region, respectively, that are related to NH_3_ twisting and the asymmetric stretch of the uranyl cation. It is notable that there is a band within the IR spectra of **Co3U2_Cl** at 948 cm^–1^ that corresponds to the band at 950 cm^–1^ within the Raman spectrum; however, there is no evidence of activation of the ν_1_ band within the IR spectrum at 809 cm^–1^. This suggests that it is not bond asymmetry driving the resulting spectral bands, but the interaction between the hydrogen donor and the uranyl oxo acceptor.

Comparing these values to previous literature results is difficult because the ν_1_ and ν_3_ bands reported may not be assigned correctly. The uranyl symmetric stretching bands for Cs_4_[UO_2_(CO_3_)_3_] and Na_4_[UO_2_(CO_3_)_3_] possess similar values to the **CoU** compounds, but other compounds range from 815 to 831 cm^–1^ (Table 2).^38,41,51,52^ Similarly, the ν_3_ band has been reported with values ranging from 843 to 912 cm^–1^ for uranyl tricarbonate species. Colmenero et al. performed DFT calculations to assess the infrared active modes of the mineral Bayerite (Mg_2_[UO_2_(CO_3_)_3_] • 18 H_2_O) and found that the band at 872 cm^–1^ could be assigned to a combination of the uranyl antisymmetric stretching vibration and water rocking modes.^46^ In addition, theoretical bands at 837 and 827 cm^–1^ are ascribed to ν_3_ stretching vibrations, carbonate out of plane bending vibrations, and twisting motions. Thus, even the presence of water within the tricarbonate system can lead to difficulties in identifying the spectral modes in these materials.

Evidence of vibrational coupling or combination modes, including the uranyl O=U=O stretch, is not without precedent, particularly with solids that contain strong intermolecular interactions. Cahill and co-workers suggested a combination mode of anharmonic resonance coupling between the benzoate ligands and the uranyl “yl” stretch of their halogenated benzoic acid and uranyl crystalline materials.^66^ In addition, Anderson and co-workers evaluated the impact on interstitial water content within the schoepite mineral phases (UO_3_•*n*H_2_O) on the resulting spectral features.^67^ Hydrogen bonding effects were found to strongly influence the symmetric stretch of each unique uranyl moiety enough to give rise to multiple stretching modes in the Raman spectra within a relatively large spectral window (810–880 cm^–1^). As noted earlier, Colmenero et al. observed that multiple uranyl features in Bayerite are coupled with water librations, water twists, and carbonate bending modes.^46^

Because our systems display significant differences in the vibrational spectra, we considered many approaches in evaluating the signals. When we base our vibrational analysis on the simple D_*∞h*_ analysis of the uranyl cation, this change in the spectra could be related to lowering of the symmetry and inducing activation. We can also consider coupled vibrational motions that can occur with the specific hydrogen bonding networks in the material as a source of the varied spectral signals. In a reductionist approach to understand the real system, we can first evaluate the simpler models and then build up the complexity to include the additional interactions, DFT calculations are well suited for this approach, and in the next section, we utilize this methodology to explore bond asymmetry without additional structural contributions and in varied coordination environments to evaluate the impacts on the vibrational modes. The first simplified set of calculations is used to delineate the impact of uranyl bond asymmetry on the vibrational features for a free uranyl cation and then for a uranyl tricarbonate species. Following those studies, we further add hydrogen bonding to the system to compare the influence of these intermolecular interactions on the resulting spectral features.

## DFT Analysis

### Forced UO_2_ Bond Asymmetry

DFT calculations are used to further evaluate the extent that uranyl bond asymmetry can impact the position of the symmetric and asymmetric stretching of the uranyl cation. To begin, we optimized the structure of a single UO_2_^2+^ cation in the absence of additional counterions. This resulted in two equivalent U=O bond lengths of 1.76 Å and ν_1_ and ν_3_ modes of 876 and 937 cm^–1^, respectively, which are within range of the previously reported computational results for the uranyl cation.^68,69^ We then varied the length of one U=O bond by 0.02 Å increments from 1.66 to 1.86 Å and fixed the second U=O bond to the optimized length of 1.76 Å. For each of the UO_2_^2+^ structures described above, a set of single-point energy calculations are performed in which the vibrational modes were calculated. The calculated values for the resulting symmetric and asymmetric stretching vibrations are listed in Table 4.

**Table 4:** DFT-Computed Vibrational Modes for the UO_2_^2+^ Unit, Where One U=O Bond Length Is Systematically Increased by 0.02 Å from 1.66 to 1.86 Å, While the Other Is Held Constant at 1.76 Å^a^

ΔU=O length (Å)	ν_1_ (cm^–1^)	ν_3_ (cm^–1^)	ν_1_/ν_3_	*k*_F_ (mdyn/Å)	*k*_12_ (mdyn/Å)
–0.10	908	1177	1.30	9.64	–1.89
–0.08	906	1120	1.24	9.08	–1.34
–0.06	903	1066	1.18	8.56	–0.88
–0.04	900	1017	1.13	8.11	–0.48
–0.02	893	973	1.09	7.69	–0.17
**0 (1.76 Å)**	**876**	**937**	**1.07**	**7.26**	**–0.03**
+0.02	844	922	1.09	6.89	–0.17
+0.04	814	948	1.16	6.85	–0.61
+0.06	761	913	1.19	6.19	–0.73
+0.08	720	911	1.27	5.89	–1.00
+0.10	681	910	1.33	5.62	–1.25

a Boldface is used to highlight the structure where the U=O bond lengths are equal at 1.76 Å.

We compare how the computed vibrational modes change as a function of bond elongation and contraction, bringing the uranyl oxo atoms closer together or further apart. When one U=O bond is elongated by 0.10 Å, the change in the ν_3_ (+195 cm^–1^) is much greater than the change in the ν_1_ (+32 cm^–1^). For both vibrational modes, there is an observed red shift. Alternatively, the contraction of one U=O bond results in a more significant change in the ν_1_ (−195 cm^–1^) compared to the ν_3_ (−27 cm^–1^); both vibrational modes exhibit a red shift as a result of asymmetric bond contraction. In general, asymmetric bond elongation results in an increase in the value of the vibrational frequencies, while bond contraction results in a decrease in the value of the vibrational frequency. When the U=O bond lengths are equivalent at 1.76 Å, the difference between the ν_1_ and ν_3_ vibrational frequencies is at a minimum. As the U=O bond length difference increases, the difference between the ν_1_ and ν_3_ vibrational frequencies increases.

The ν_1_**/**ν_3_ ratio is reported and compared to previous results where it is used to evaluate the impact of the interaction force constant within the uranyl bond.^19,20,38,70^ Vibrational modes associated with the uranyl cation are also related to the force constant (*k*_1_) and the interaction force constant (*k*_12_). If we consider a simple valence force field and assume harmonic vibrational for the linear ion, then the interaction force constant can be omitted and the relationship between ν_1_ and ν_3_ can be written as:
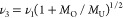
1where *M*_O_ and *M*_U_ represent the mass of the O and U atoms, respectively. This leads to a ν_3_**/**ν_1_ of 1.065, which is identical to that calculated for our symmetric uranyl bond (1.07). When the interaction force constant is included, then the ν_3_**/**ν_1_ ratio will increase or decrease depending on the overall sign of the *k*_12_. In the case of our bond asymmetry, we note that ν_3_**/**ν_1_ increases, which indicates that the interaction force constant decreases. This can be observed in the calculated *k*_12_ values, where more negative values are obtained when one bond is either lengthened or shorted to induce asymmetry. The trend is different for k_1_, where the value is dependent on the length of the bond, with shorter distances related to stronger force constants.

Schnaars and Wilson evaluated force constants for a series of uranyl tetrachloride compounds, and these compare well to the results associated with our computed uranyl cation.^71,72^ At a symmetric bond distance of 1.76 Å, the theoretical *k*_1_ was calculated at 7.26 mdyn/Å and decreased to 5.62 mdyn/Å with a bond elongation of 0.1 Å. This is well within the range that has been experimentally observed within the tetrachloride system (6.39–6.74 mdyn/Å).^73^ Additionally, the *k*_12_ was observed between −0.10 and −0.53 mdyn/Å, and this matches well with a small negative value that was obtained from DFT analysis. It is interesting to note that the ν_3_/ν_1_ ratio for the uranyl tetrachloride compounds ranges from 1.08 to 1.10, which is slightly higher than the value assumed for minimal contribution of the *k*_12_ (1.065). This suggests that the small contribution from the interaction force can be observed by utilizing the vibrational band ratios.

A similar approach was followed for the [UO_2_(CO_3_)_3_]^4–^ structure. The initial coordinates were obtained from the experimental crystal structure. The [UO_2_(CO_3_)_3_]^4–^ was first subjected to geometry optimization, where the U=O bonds optimized to equivalent lengths of 1.82 Å. Visualization of the vibrational modes for the optimized [UO_2_(CO_3_)_3_]^4–^ structure displayed two ν_1_ modes at 789 (ν_1a_) and 717 (ν_1b_) cm^–1^ and a ν_3_ mode at 830 cm^–1^. The two ν_1_ modes display coupling of the UO_2_ symmetric stretch and the ν_2_ wagging motion of the bound CO_3_^2–^ group. The ν_1a_ band displays symmetric uranyl contraction coupled to an inward ν_2_ CO_2_ wag, whereas ν_1b_ consists of a uranyl contraction coinciding with an outward ν_2_ wag motion of the bound CO_3_^2–^ ligands.

To investigate the effects of U=O bond asymmetry on the vibrational modes in [UO_2_(CO_3_)_3_]^4–^, a series of calculations at fixed geometry was carried out. The interatomic separation of one of the U=O was varied by 0.02 Å from 1.82 to 1.72 Å. We chose 1.82 Å as the longest distance that is observed for the [UO_2_(CO_3_)_3_]^4–^ with symmetric U=O bond lengths. These calculations allow for the comparison of the change in the vibrational modes with the presence of ligands in the equatorial plane, but without the interaction of additional species.

The ν_1_ and ν_3_ vibrational frequencies were monitored as the extent of the bond asymmetry increased along the series (Table 3). Comparing the ν_1a_ and ν_1b_ modes, we observe an overall red shift of 9 or 11 cm^–1^, respectively, when the U=O lengths differ by 0.1 Å. When the asymmetric U=O bond contraction for the [UO_2_(CO_3_)_3_]^4–^ complex differs by 0.10 Å, there is a more significant red shift in the ν_3_ (+188 cm^–1^) than for the ν_1_ (+9 cm^–1^), which is similar to the free UO_2_^2+^ system.

We evaluated the ratio of the symmetric and asymmetric bands to provide further insight into the system (Table 5). Inducing U=O bond asymmetry of the uranyl tricarbonate complex does not change the ν_1a_/ν_1b_ ratio, which remains constant at 1.10 throughout the entire range of tested asymmetry values. Both the ν_3_/ν_1a_ and ν_3_/ν_1b_ ratios increase with increasing U=O bond asymmetry. For ν_3_/ν_1a_, the ratio is similar to the harmonic model (1.05) when the bonds are both at 1.82 Å and increase to 1.26 when the bond difference is −0.1 Å. For the ν_3_/ν_1b_ ratio, it begins with a larger value (1.16) because of a larger energy difference between the modes and increases to 1.40 with induced asymmetry.

**Table 5:** DFT-Computed Vibrational Modes for the [UO_2_(CO_3_)_3_]^4–^ Unit, Where One U=O Bond Length Is Systematically Decreased by 0.02 Å and the Other Is Held Constant at 1.82 Å^a^

ΔU=O length (Å)	ν_1-a,_ ν_1-b_ (cm^–1^)	Δν_1_	ν_1-a_/ν_1-b_	ν_3_ (cm^–1^)	ν_3_/ν_1-a_	ν_3_/ν_1-b_
–0.10	802, 726	76	1.10	1018	1.26	1.40
–0.08	801, 725	76	1.10	973	1.21	1.34
–0.06	800, 724	76	1.11	930	1.16	1.28
–0.04	803, 733	70	1.10	914	1.14	1.24
–0.02	795, 720	75	1.10	855	1.08	1.19
**0 (1.82 Å)**	**789, 717**	**72**	**1.10**	**830**	**1.05**	**1.16**

a Boldface is used to highlight the structure where the U=O bond lengths are equal at 1.82 Å.

### Counter-Cation Interactions

**Figure 4:**
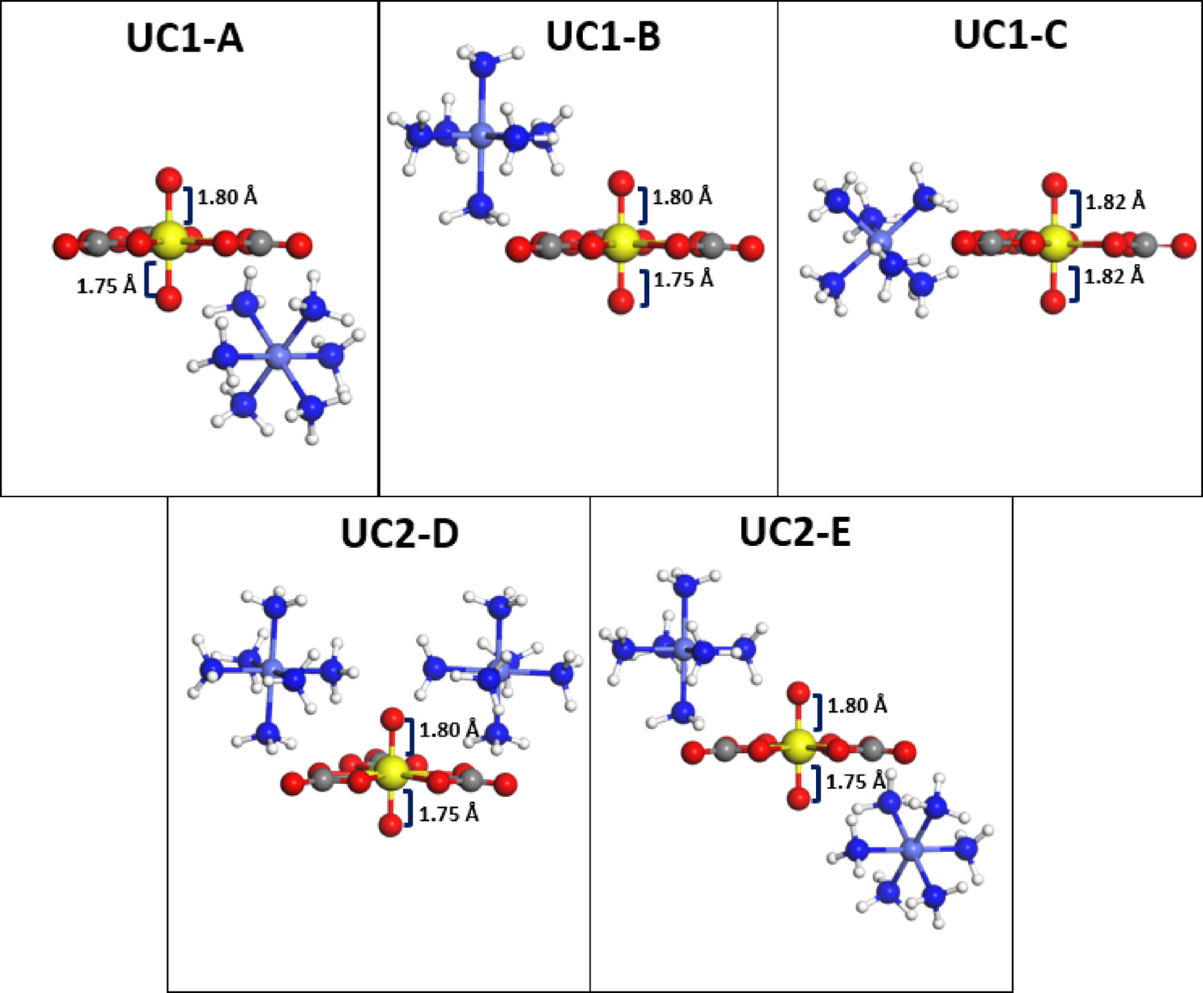
Ball and stick representations of the molecular models used in the DFT calculations of UO_2_(CO_3_)_3_^4–^ interacting with [Co(NH_3_)_6_]^3+^ cations, where the number and position of the cation are altered. Models UC1-A, -B, and -C contain one [Co(NH_3_)_6_]^3+^ cation, whereas UC2-D and -E contain two counter cations. Uranium, oxygen, carbon, nitrogen, cobalt, and hydrogen are depicted as yellow, red, gray, dark blue, light blue, and white spheres, respectively.

The next series of DFT calculations were performed on systems that varied the position and number of the [Co(NH_3_)_6_]^3+^ cation around the uranyl carbonate complex. Isolated uranyl carbonate (UC) was optimized, and then either one (UC1-A, UC1-B, or UC1-C) or two (UC2-D and UC2-E) [Co(NH_3_)_6_]^3+^ cations were placed around the uranyl tricarbonate anions in locations obtained from the crystallographic information files (Figure 4). The geometry of the isolated [UO_2_(CO_3_)_3_]^4–^ complex (denoted as UC) was optimized and included here for comparison to the models that contained the cobalt hexamine counterion. As previously mentioned, bond lengths in the isolated [UO_2_(CO_3_)_3_]^4–^ molecular complex are symmetric, with U=O bonds of 1.82 Å. Bonding to the carbonate anions leads to U–O_c_ equatorial distances of 2.45 Å and U–C interatomic values of 2.91 Å. These theoretical bond distance values agree well with other values observed by Reeder et al. and Ikeda et al.^74,75^Figure 4 also depicts the interactions between the [Co(NH_3_)_6_]^3+^ cation with asymmetric uranyl bonds. In UC1-A, the cobalt hexamine interacts with the O atoms associated with the shorter U=O bond (1.75 Å) with a D–H···A distance of 2.91 Å. Alternatively, UC-B shows the interaction of the [Co(NH_3_)_6_]^3+^ cation with the oxo group of the longer U=O distance (1.80 Å) and D–H···A distance of 3.51 Å. Geometry optimization of either UC1-A or UC1-B results in the structure shown in UC1-C, where the cobalt hexamine has moved from its position near the axial oxo groups to the equatorial plane, where it can engage in H-bonding interactions with the carbonate anions. The optimized structure has identical bond U=O bond lengths (1.82 Å) to that observed for the isolated uranyl tricarbonate anion. UC2-D and UC2-E depict the interaction between the uranyl tricarbonate and two [Co(NH_3_)_6_]^3+^ cations at different positions around the metal complex. In both cases, the uranyl bond is modeled as asymmetric to further understand the impact of the intermolecular H-bonding on the vibrational modes within these complexes. UC2-D is modeled with the two cobalt hexamine cations engaged in interactions with the longer U=O (1.80 Å), with D–H···A distances of 3.63 Å. UC2-E is modeled with the one cobalt hexamine cations engaged in interactions with each uranyl oxo, with D–H···A distances of 3.63 Å to the longer U=O (1.80 Å) and D–H···A of 2.91 Å to the shorter U=O (1.75 Å).

The symmetric and asymmetric uranyl stretching modes were identified in all three structures, and associated frequency values are reported in Table 6. Only the symmetric/asymmetric modes obtained from structures that exhibit atomic displacements greater than 0.01 Å for the uranyl are reported herein. As mentioned previously, the isolated [UO_2_(CO_3_)_3_]^4–^ unit (UC) has two ν_1_ modes at 717 and 789 cm^–1^ and a ν_3_ mode at 830 cm^–1^, where the presence of multiple ν_1_ modes is related to coupling between the uranyl stretching modes and the carbonate motions. Each mode will be described in detail for a given structure to show how the uranyl modes shift as a function of counter cation position and number.

**Table 6:** DFT-Calculated Uranyl Active Vibrational Modes for UC Structures Containing Cobalt Hexamine Cation(s) and an Isolated Molecular Complex for Comparison^a^

structure	U=O lengths (Å)	ν_1-a_, ν_1-b_ (cm^–1^)	Δν_1_	ν_3_ (cm^–1^)	ν_1-a_/ν_1-b_	ν_3_/ν_1-a_	ν_3_/ν_1-b_
isolated UC	1.82, 1.82	789, 717	72	830	1.10	1.05	1.15
isolated UC/asymmetric	1.82, 1.78	800, 724	76	930	1.11	1.16	1.28
UC1-A	1.80, 1.75(O···H)	861, 738	123	960	1.17	1.15	1.30
UC1-B	1.80(O···H), 1.75	868, 716	145	986	1.21	1.13	1.37
UC1-C	1.82, 1.82	803, 724	79	835	1.11	1.04	1.15
UC2-D	1.80(O···H), 1.75	871, 738	133	960	1.18	1.10	1.30
UC2-E	1.80(O···H), 1.75(O···H)	864, 736	128	961	1.17	1.11	1.30

a Uranyl bond lengths engaged in a H-bond are denoted with (O···H) following the value.

With the presence of hydrogen bonding, all ν_1_ and ν_3_ uranyl symmetric stretching features are shifted to higher wavenumbers compared to the isolated forms. The UC1-A interaction geometry has ν_1a_ and ν_1b_ modes located at 738 and 861 cm^–1^ and one major ν_3_ mode at 960 cm^–1^. With the presence of the [Co(NH_3_)_6_]^3+^ cation, additional concerted motions are noted as the mode at 861 cm^–1^ shows coupling between the uranyl stretching mode, inward ν_2_ CO_3_^2–^ wag, and the twisting of the cobalt hexamine cation. Similarly, the band at 960 cm^–1^ exhibits coupled uranyl and cobalt hexamine motions. Structure UC1-B indicates that the ν_1b_ mode is predicted to be at 716 cm^–1^ and ν_1a_ band located at 868 cm^–1^ is coupled to the cobalt hexamine through hydrogen bonding.

Structure UC1-C has equivalent U=O bond lengths, and the vibrational modes can be compared to the [(UO_2_)(CO_3_)_3_]^4–^ complex. Like the isolated molecule, the symmetric ν_1_ modes in UC1-C occur at 724 and 803 cm^–1^. Both modes exhibit identical motions of coupled uranyl ν_1_ and carbonate ν_2_ modes with minimal hydrogen-bonding contributions between the O_yl_ atoms and the cobalt hexamine that are now located along the equatorial plane. The asymmetric ν_3_ uranyl mode occurs at 835 cm^–1^ and contributions from the carbonate ν_3_ mode.

Comparatively, structures UC1-A and UC1-B have more similar vibrational modes to one another than to either structure UC1-C or the isolated UC. These two structures share the same unequal uranyl axial bond lengths (1.75 and 1.80 Å) and partake in hydrogen-bonding interactions with the counter cation. Structures UC1-A and UC1-B have ν_1_ modes at 861 and 868 cm^–1^, respectively, which are not present in the Isolated UC or Structure UC1-C. Alternatively, both optimized geometries of structure UC1-C and the isolated UC model have the same uranyl axial bond lengths (1.82 Å) and similar vibrational modes. For example, both structure UC1-C and the isolated UC models have a symmetric mode near or at 717 cm^–1^. Additionally, the isolated UC complex has an asymmetric ν_3_ mode at 830 cm^–1^, which resemble the mode for structure UC1-C at 835 cm^–1^. The asymmetric modes for structures UC1-A and UC1-B are blue-shifted to frequencies greater than 960 cm^–1^ and are not present in the 830–850 cm^–1^ range.

The ν_1_ modes vibrational modes for structures UC2-D and E are analogous to the bands associated with the UC1-A. Both the ν _1b_ modes (738 and 736 cm^–1^) can be linked to uranyl bond displacements coupled to carbonate ν_2_ motions, with minor hydrogen breathing motions from the cobalt hexamine cations. UC2-D has a second ν_1_ mode at 871 cm^–1^, which is blue-shifted relative to the same band in UC2-E at 864 cm^–1^. Both structures display ν_3_ modes at 960/961 cm^–1^, in which the uranyl oxo engages in hydrogen-bonding interactions with cobalt hexamine groups.

From the vibrational bands, we can evaluate the impact of hydrogen bonding on the ν_1_ and ν_3_ ratios. Unlike the isolated system where the ν_1a_/ν_1b_ ratio was constant at 1.10, the series including the cobalt hexamine shows a range of higher values 1.17–1.21. This may be caused by an inequivalent hydrogen bonding network that contributes to the combination mode (oxo groups and carbonate anions), which impacts these bands differently, manifesting in nonmonatomic changes in the ratio. The ν_3_/ν_1a_ and the ν_3_/ν_1b_ ratios are larger than those observed for the isolated system with symmetric U=O bonds, but these values are within the region for isolated [UO_2_(CO_3_)]^4–^ complexes with induced bond asymmetry. This suggests that the impact of the hydrogen bonding network combined with the asymmetry can lead to larger values for this ratio which would need to be considered if using ratios to confirm vibrational assignment of uranyl modes.

### Comparison of Theoretical and Experimental Results

If we compare the DFT vibrational analysis to the spectroscopic measurements of the **CoU** compounds, we find that we can utilize band ratios to help confirm our spectral assignment. Previous combined experimental studies with computational efforts found similar success in assignments of challenging vibrational spectra.^76−78^ The ν_1-a_/ν_1-b_ ratios for all CoU compounds were calculated to be 1.10–1.11, which is the same as what is expected for a symmetric uranyl bond. There are multiple concerted motions that contain the ν_1-a_ or ν_3_ bands, so determining the accurate ratio is not straightforward. This speaks to the impact that the hydrogen bonding network has on the asymmetric stretch (ν_3_). DFT calculations demonstrated that hydrogen bonding between the uranyl oxo and a hydrogen donor can cause a blue shift in the ν_1_ and ν_3_ uranyl stretching bands. We do not see evidence of this perturbation for the symmetric ν_1_ stretching bands but do see significant concerted motions of the NH_3_ and uranyl oxo groups to create multiple ν_3_ asymmetric modes that are shifted to higher wavenumbers, with respect to the isolated complex. This demonstrates that the Raman spectra are less impacted by the hydrogen bonding network than the infrared spectra in our system.

## Conclusions

The uranyl tricarbonate anion was crystallized with the cobalt hexamine cation to form four different solid-state materials ([Co(NH_3_)_6_]_4_[UO_2_(CO_3_)_3_]_3_H_2_O_11.67_ (**Co4U3**), [Co(NH_3_)_6_]_3_[UO_2_(CO_3_)_3_]_2_Cl H_2_O_7.5_ (**Co3U2_Cl**), [Co(NH_3_)_6_]_2_[UO_2_(CO_3_)_3_]Cl_2_ (**Co2U_Cl**), and [Co(NH_3_)_6_]_2_[UO_2_(CO_3_)_3_]CO_3_ (**Co2U_CO_3_**). Structural analysis of the compounds revealed no significant perturbation of the uranyl bonds but displayed differences in the hydrogen bonding network within these compounds. Raman and infrared spectroscopy revealed different spectral features that were related to combination modes associated with the hydrogen bonding networks. DFT calculations were performed to evaluate the impacts of bond perturbation compared to changes in the hydrogen bonding network. Overall, bond perturbation led to an increased ν_3_/ν_1_ ratio, indicating that the interaction force constant (*k*_12_) should be considered for this system. Addition of hydrogen bonding to the uranyl oxo groups led to a blue shift in the vibrational features, and these interactions impact the ν_3_ band more significantly than the ν_1_.

Understanding the complex spectral features related to hydrogen bonding networks in solid and solution U(VI) phases will provide insights into uranyl bond modification, improve our knowledge of uranyl speciation in aqueous solutions, and enhance the use of these methodologies in sensing and nuclear forensics capabilities. This study demonstrates that combination bands within uranyl solids can lead to significant complexity within the vibrational spectra and should be further evaluated to provide a detailed understanding of these features. Additional studies should also focus on providing relationships between spectral bands that can be further utilized to identify specific U(VI) phases and a more descriptive understanding of the interactions that take place between the actinyl oxo groups and neighboring molecules and ions.

## Supporting Information Available

Bond distances for solid-state compounds, hydrogen bonding tables, thermal ellipsoid of asymmetric units, powder X-ray diffractograms, and additional details of the DFT calculations (PDF)

## References

[ref1] Guerra W. D.; Odella E.; Secor M.; Goings J. J.; Urrutia M. N.; Wadsworth B. L.; Gervaldo M.; Sereno L. E.; Moore T. A.; Moore G. F.; Hammes-Schiffer S.; Moore A. L. Role of Intact Hydrogen-Bond Networks in Multiproton-Coupled Electron Transfer. J. Am. Chem. Soc. 2020, 142, 21842–21851. 10.1021/jacs.0c10474. 33337139

[ref2] Shokri A.; Wang Y.; O’Doherty G. A.; Wang X.-B.; Kass S. R. Hydrogen-Bond Networks: Strengths of Different Types of Hydrogen Bonds and An Alternative to the Low Barrier Hydrogen-Bond Proposal. J. Am. Chem. Soc. 2013, 135, 17919–17924. 10.1021/ja408762r. 24188017

[ref3] Kamali N.; Aljohani M.; McArdle P.; Erxleben A. Hydrogen Bonding Networks and Solid-State Conversions in Benzamidinium Salts. Cryst. Growth Des. 2015, 15, 3905–3916. 10.1021/acs.cgd.5b00529.

[ref4] Arunan E.; Desiraju G. R.; Klein R. A.; Sadlej J.; Scheiner S.; Alkorta I.; Clary D. C.; Crabtree R. H.; Dannenberg J. J.; Hobza P.; Kjaergaard H. G.; Legon A. C.; Mennucci B.; Nesbitt D. J. Definition of the hydrogen bond (IUPAC Recommendations 2011). Pure Appl. Chem. 2011, 83, 1637–1641. 10.1351/PAC-REC-10-01-02.

[ref5] Desiraju G. R. Hydrogen bonds and other intermolecular interactions in organometallic crystals. J. Chem. Soc., Dalton Trans. 2000, 21, 3745–3751. 10.1039/B003285I.

[ref6] Desiraju G. R.; Steiner T. The Weak Hydrogen Bond: In Structural Chemistry and Biology: 9 (International Union of Crystallography Monographs on Crystallography); OUP Oxford, 2001.

[ref7] Anderson N. H.; Xie J.; Ray D.; Zeller M.; Gagliardi L.; Bart S. C. Elucidating bonding preferences in tetrakis (imido) uranate (VI) dianions. Nat. Chem. 2017, 9, 850–855. 10.1038/nchem.2767. 28837176

[ref8] Burns P. C.; Finch R. J. Uranium: mineralogy, geochemistry, and the environment; Walter de Gruyter GmbH & Co KG, 2018; Vol. 38.

[ref9] Choppin G.; Liljenzin J.-O.; Rydberg J.; Ekberg C. The Actinide and Transactinide Elements. In Radiochemistry and Nuclear Chemistry, 4th ed.; Choppin G.; Liljenzin J.-O.; Rydberg J.; Ekberg C., Eds.; Academic Press: Oxford, 2013; pp 405–444.

[ref10] Loiseau T.; Mihalcea I.; Henry N.; Volkringer C. The crystal chemistry of uranium carboxylates. Coord. Chem. Rev. 2014, 266–267, 69–109. 10.1016/j.ccr.2013.08.038.

[ref11] Andrews M. B.; Cahill C. L. Uranyl Bearing Hybrid Materials: Synthesis, Speciation, and Solid-State Structures. Chem. Rev. 2013, 113, 1121–1136. 10.1021/cr300202a. 22881287

[ref12] Watson L. A.; Hay B. P. Role of the Uranyl Oxo Group as a Hydrogen Bond Acceptor. Inorg. Chem. 2011, 50, 2599–2605. 10.1021/ic102448q. 21291200

[ref13] Fortier S.; Hayton T. W. Oxo ligand functionalization in the uranyl ion (UO22+). Coord. Chem. Rev. 2010, 254, 197–214. 10.1016/j.ccr.2009.06.003.

[ref14] Arnold P. L.; Patel D.; Wilson C.; Love J. B. Reduction and selective oxo group silylation of the uranyl dication. Nature 2008, 451, 315–317. 10.1038/nature06467. 18202653

[ref15] Love J. B. A macrocyclic approach to transition metal and uranyl Pacman complexes. Chem. Commun. 2009, 22, 3154–3165. 10.1039/B904189C. 19587900

[ref16] Jones G. M.; Arnold P. L.; Love J. B. Oxo–Group-14-Element Bond Formation in Binuclear Uranium(V) Pacman Complexes. Chem. – Eur. J. 2013, 19, 10287–10294. 10.1002/chem.201301067. 23794441

[ref17] Pakiari A. H.; Eskandari K. The chemical nature of very strong hydrogen bonds in some categories of compounds. J. Mol. Struct.: THEOCHEM 2006, 759, 51–60. 10.1016/j.theochem.2005.10.040.

[ref18] Lu G.; Haes A. J.; Forbes T. Z. Detection and identification of solids, surfaces, and solutions of uranium using vibrational spectroscopy. Coord. Chem. Rev. 2018, 374, 314–344. 10.1016/j.ccr.2018.07.010. 30713345 PMC6358285

[ref19] Bullock J. I.; Parrett F. W. The low frequency infrared and Raman spectroscopic studies of some uranyl complexes: the deformation frequency of the uranyl ion. Can. J. Chem. 1970, 48, 3095–3097. 10.1139/v70-520.

[ref20] Bullock J. I. Raman and infrared spectroscopic studies of the uranyl ion: the symmetric stretching frequency, force constants, and bond lengths. J. Chem. Soc. A 1969, 0, 781–784. 10.1039/J19690000781.

[ref21] Matar S. F. Lattice anisotropy, electronic and chemical structures of uranyl carbonate, UO_2_CO_3_, from first principles. Chem. Phys. 2010, 372, 46–50. 10.1016/j.chemphys.2010.04.020.

[ref22] Thiyagarajan S.; Rajan S. S.; Gautham N. Cobalt hexammine induced tautomeric shift in Z-DNA: the structure of d(CGCGCA)*d(TGCGCG) in two crystal forms. Nucleic Acids Res. 2004, 32, 5945–5953. 10.1093/nar/gkh919. 15534365 PMC528804

[ref23] Tajmir-Riahi H. A.; Naoui M.; Ahmad R. The effects of cobalt-hexammine and cobalt-pentammine cations on the solution structure of calf-thymus DNA. DNA condensation and structural features studied by FTIR difference spectroscopy. J. Biomol. Struct. Dyn. 1993, 11, 83–93. 10.1080/07391102.1993.10508711. 8216950

[ref24] Mitsuhashi R.; Suzuki T.; Hosoya S.; Mikuriya M. Hydrogen-Bonded Supramolecular Structures of Cobalt(III) Complexes with Unsymmetrical Bidentate Ligands: mer/fac Interconversion Induced by Hydrogen-Bonding Interactions. Cryst. Growth Des. 2017, 17, 207–213. 10.1021/acs.cgd.6b01438.

[ref25] Krestou A.; Panias D. Uranium (VI) speciation diagrams in the UO22+/CO320/H2O system at 25 C. Eur. J. Miner. Process. Environ. Prot. 2004, 4, 113–129.

[ref26] La Plante E. C.; Simonetti D. A.; Wang J.; Al-Turki A.; Chen X.; Jassby D.; Sant G. N. Saline Water-Based Mineralization Pathway for Gigatonne-Scale CO_2_ Management. ACS Sustainable Chem. Eng. 2021, 9, 1073–1089. 10.1021/acssuschemeng.0c08561.

[ref27] Sheldrick G. M. APEX3; Bruker AXS: Madison, WI, 2015.

[ref28] Sheldrick G. M. Acta Crystallogr., Sect. A: Found. Crystallogr. 2008, 64, 112–122. 10.1107/S0108767307043930. 18156677

[ref29] Origin(Pro), 2019b; OriginLab Corporation.

[ref30] Becke A. D. Density-functional thermochemistry. III. The role of exact exchange. J. Chem. Phys. 1993, 98, 5648–5652. 10.1063/1.464913.

[ref31] GmbH; TUROBOMOLE V7.2; University of Karlsruhe: Karlsuhe, Germany, 2007.

[ref32] Cao X.; Dolg M.; Stoll H. Valence basis sets for relativistic energy-consistent small-core actinide pseudopotentials. J. Chem. Phys. 2003, 118, 487–496. 10.1063/1.1521431.

[ref33] Dolg M. Chapter 14 - Relativistic Effective Core Potentials. In Theoretical and Computational Chemistry; Schwerdtfeger P., Ed.; Elsevier: 2002; Vol. 11, pp 793–862.

[ref34] Küchle W.; Dolg M.; Stoll H.; Preuss H. Energy-adjusted pseudopotentials for the actinides. Parameter sets and test calculations for thorium and thorium monoxide. J. Chem. Phys. 1994, 100, 7535–7542. 10.1063/1.466847.

[ref35] Klamt A.; Schürmann G. COSMO: a new approach to dielectric screening in solvents with explicit expressions for the screening energy and its gradient. J. Chem. Soc., Perkin Trans. 2 1993, 5, 799–805. 10.1039/P29930000799.

[ref36] Jaquet R.; Haeuseler H. Vibrational analysis of the H_4_I_2_O_10_^2–^ ion in CuH_4_I_2_O_10_·6H_2_O. J. Raman Spectrosc. 2008, 39, 599–606. 10.1002/jrs.1889.

[ref37] Graziani R.; Bombieri G.; Forsellini E. Crystal structure of tetra-ammonium uranyl tricarbonate. J. Chem. Soc., Dalton Trans. 1972, 19, 2059–2061. 10.1039/DT9720002059.

[ref38] Čejka J. 12. Infrared Spectroscopy and Thermal Analysis of the Uranyl Minerals. In Uranium; De Gruyter, 2018; pp 521–622.

[ref48] Novitskiy G. G.; Komyak A. I.; Umreyko D. S. Uranyl compounds, Vol 2: Atlas of spectra; Beloruss State University: Minsk, 1981; p 216.

[ref58] Mishkevich V. I.; Grigoriev M. S.; Fedosseev A. M.; Moisy P. Guanidinium dioxidobis(picolinato-κ(2)N,O)(picolinato-κO)uranate(VI). Acta Crystallogr., Sect. E: Struct. Rep. Online 2012, 68, m1243 10.1107/S1600536812035465. PMC3470132 23125576

[ref39] Allen P. G.; Bucher J. J.; Clark D. L.; Edelstein N. M.; Ekberg S. A.; Gohdes J. W.; Hudson E. A.; Kaltsoyannis N.; Lukens W. W.; Neu M. P.; Palmer P. D.; Reich T.; Shuh D. K.; Tait C. D.; Zwick B. D. Multinuclear NMR, Raman, EXAFS, and X-ray diffraction studies of uranyl carbonate complexes in near-neutral aqueous solution. X-ray structure of [C(NH_2_)_3_]_6_[(UO_2_)_3_(CO_3_)_6_].cntdot.6.5H_2_O. Inorg. Chem. 1995, 34, 4797–4807. 10.1021/ic00123a013.

[ref40] Reed W. A.; Oliver A. G.; Rao L. Tetrakis(tetramethylammonium) tricarbonatodioxidouranate octahydrate. Acta Crystallogr., Sect. C: Cryst. Struct. Commun. 2011, 67, m301–m303. 10.1107/S0108270111032641. 21881176

[ref79] Li Y.; Krivovichev S. V.; Burns P. C. The crystal structure of Na_4_ (UO_2_)(CO_3_)_3_ and its relationship to schrockingerite. Mineral. Mag. 2001, 65 (2), 297–304. 10.1180/002646101550262.

[ref41] Frost R. L.; Erickson K. L.; Weier M. L.; Carmody O.; Čejka J. Raman spectroscopic study of the uranyl tricarbonate mineral liebigite. J. Mol. Struct. 2005, 737, 173–181. 10.1016/j.molstruc.2004.10.033.

[ref43] Coda A.; Della Giusta A.; Tazzoli V. The structure of synthetic andersonite, Na2Ca [UO_2_ (CO_3_)_3_]. *x*H_2_O (*x* ≃ 5.6). Acta Crystallogr., Sect. B: Struct. Crystallogr. Cryst. Chem. 1981, 37, 1496–1500. 10.1107/S0567740881006432.

[ref80] Driscoll R. J. P.; Wolverson D.; Mitchels J. M.; Skelton J. M.; Parker S. C.; Molinari M.; Khan I.; Geeson D.; Allen G. C. A Raman spectroscopic study of uranyl minerals from Cornwall, UK. RSC Adv. 2014, 4, 59137–59149. 10.1039/C4RA09361E.

[ref44] Hughes Kubatko K.-A.; Burns P. C. The Rb analogue of grimselite, Rb_6_Na_2_[(UO_2_)(CO_3_)_3_]_2_(H_2_O). Acta Crystallogr., Sect. C: Cryst. Struct. Commun. 2004, 60, i25–i26. 10.1107/S0108270103028312. 15004352

[ref45] Olds T. A.; Plášil J.; Kampf A. R.; Dal Bo F.; Burns P. C. Paddlewheelite, a New Uranyl Carbonate from the Jáchymov District, Bohemia, Czech Republic. Minerals 2018, 8, 1–16. 10.3390/min8110511.

[ref42] Mayer H.; Mereiter K. Synthetic bayleyite, Mg_2_[UO_2_(CO_3_)_3_]·18H_2_O: Thermochemistry, crystallography and crystal structure. Tschermaks Mineral. Petrogr. Mitt. 1986, 35, 133–146. 10.1007/BF01140845.

[ref46] Colmenero F.; Plášil J.; Škácha P. The magnesium uranyl tricarbonate octadecahydrate mineral, bayleyite: Periodic DFT study of its crystal structure, hydrogen bonding, mechanical properties and infrared spectrum. Spectrochim. Acta, Part A 2020, 234, 118216 10.1016/j.saa.2020.118216. 32171155

[ref47] Colmenero F. Thermodynamic properties of the uranyl carbonate minerals roubaultite, fontanite, widenmannite, grimselite, čejkaite and bayleyite. Inorg. Chem. Front. 2020, 7, 4160–4179. 10.1039/D0QI01019G.

[ref81] Anderson A.; Chieh C.; Irish D. E.; Tong J. P. K. An X-ray crystallographic, Raman, and infrared spectral study of crystalline potassium uranyl carbonate, K4UO2(CO3)3.. Can. J. Chem. 1980, 58 (16), 1651–1658. 10.1139/v80-264.

[ref49] Plášil J.; Šejka J.; Sejkora J.; Hloušek J.; Škoda R.; Novák M.; Dušek M.; Císařová I.; Němec I.; Ederová J. Linekite, K_2_Ca_3_[(UO_2_)(CO_3_)_3_]_2_.8H_2_O, a new uranyl carbonate mineral from Jachymov, Czech Republic. J. Geosci. 2017, 62, 201–213. 10.3190/jgeosci.241.

[ref50] Chernorukov N. G.; Mikhailov Y. N.; Knyazev A. V.; Kanishcheva A. S.; Zamkovaya E. V. Synthesis and Crystal Structure of Rubidium Uranyltricarbonate. Russ. J. Coord. Chem. 2005, 31, 364–367. 10.1007/s11173-005-0105-3.

[ref51] Gorbenko-Germanov D. S. Vibrational specta of alkali metal uranyl tricarbonates and uranyl trinitrates; Academy of Sciences: Moscow, 1962; Vol. I.

[ref52] Krivovichev S. V.; Burns P. C. Synthesis and Crystal Structure of Cs_4_[UO_2_(CO_3_)_3_]. Radiochemistry 2004, 46, 12–15. 10.1023/B:RACH.0000024627.56487.31.

[ref53] Mereiter K. The crystal structure of Liebigite, Ca_2_UO_2_(CO_3_)_3_·∼ 11H_2_O. Tschermaks Mineral. Petrogr. Mitt. 1982, 30, 277–288. 10.1007/BF01087173.

[ref54] Kampf A. R.; Olds T. A.; Plášil J.; Burns P. C.; Marty J. Natromarkeyite and pseudomarkeyite, two new calcium uranyl carbonate minerals from the Markey mine, San Juan County, Utah, USA. Mineral. Mag. 2020, 84, 753–765. 10.1180/mgm.2020.59.

[ref55] Mereiter K. Synthetic swartzite, CaMg[UO_2_(CO_3_)_3_]12H_2_O, and its strontium analogue, SrMg[UO_2_(CO_3_)_3_]12H_2_O: Crystallography and crystal structure. Neues Jahrb. Mineral., Monatsh. 1986, 11, 481–492.

[ref56] Rofail N. Infrared and X-ray diffraction spectra of ammonium uranyl carbonate. Mater. Chem. Phys. 1994, 36, 241–245. 10.1016/0254-0584(94)90036-1.

[ref57] Gurzhiy V. V.; Kalashnikova S. A.; Kuporev I. V.; Plášil J. Crystal Chemistry and Structural Complexity of the Uranyl Carbonate Minerals and Synthetic Compounds. Crystals 2021, 11, 704 10.3390/cryst11060704.

[ref59] Jeffrey G. A. An Introduction to Hydrogen Bonding; Oxford University Press, 1997.

[ref60] Jeffrey G. A.; Saenger W. Hydrogen Bonding in Biological Structures; Springer: Berlin Heidelberg, 2012.

[ref61] Zhang Y.; Collison D.; Livens F. R.; Helliwell M.; Heatley F.; Powell A. K.; Wocadlo S.; Eccles H. Synthesis and characterisation of uranyl substituted malonato complexes: Part II: 13C CPMAS NMR spectroscopy related to structural diversity. Polyhedron 2002, 21, 81–96. 10.1016/S0277-5387(01)00965-2.

[ref62] Zhang Y.; Collison D.; Livens F. R.; Helliwell M.; Eccles H.; Tinker N. Structural studies on monomeric and dimeric uranyl bis(dimethylmalonato)complexes. J. Alloys Compd. 1998, 271–273, 139–143. 10.1016/S0925-8388(98)00041-3.

[ref63] Clark D. L.; Conradson S. D.; Donohoe R. J.; Keogh D. W.; Morris D. E.; Palmer P. D.; Rogers R. D.; Tait C. D. Chemical Speciation of the Uranyl Ion under Highly Alkaline Conditions. Synthesis, Structures, and Oxo Ligand Exchange Dynamics. Inorg. Chem. 1999, 38, 1456–1466. 10.1021/ic981137h.

[ref64] Vallet V.; Wahlgren U.; Schimmelpfennig B.; Moll H.; Szabó Z.; Grenthe I. Solvent Effects on Uranium(VI) Fluoride and Hydroxide Complexes Studied by EXAFS and Quantum Chemistry. Inorg. Chem. 2001, 40, 3516–3525. 10.1021/ic001405n. 11421700

[ref65] Sonnenberg J. L.; Hay P. J.; Martin R. L.; Bursten B. E. Theoretical Investigations of Uranyl–Ligand Bonding: Four- and Five-Coordinate Uranyl Cyanide, Isocyanide, Carbonyl, and Hydroxide Complexes. Inorg. Chem. 2005, 44, 2255–2262. 10.1021/ic048567u. 15792460

[ref66] Ridenour J. A.; Schofield M. H.; Cahill C. L. Structural and Computational Investigation of Halogen Bonding Effects on Spectroscopic Properties within a Series of Halogenated Uranyl Benzoates. Cryst. Growth Des. 2020, 20, 1311–1318. 10.1021/acs.cgd.9b01567.

[ref67] Kirkegaard M. C.; Niedziela J. L.; Miskowiec A.; Shields A. E.; Anderson B. B. Elucidation of the structure and vibrational spectroscopy of synthetic metaschoepite and its dehydration product. Inorg. Chem. 2019, 58, 7310–7323. 10.1021/acs.inorgchem.9b00460. 31099558

[ref68] Lewis A. J.; Yin H.; Carroll P. J.; Schelter E. J. Uranyl-oxo coordination directed by non-covalent interactions. Dalton Trans. 2014, 43, 10844–10851. 10.1039/C4DT00763H. 24894554

[ref69] Bjorklund J. L.; Pyrch M. M.; Basile M. C.; Mason S. E.; Forbes T. Z. Actinyl-cation interactions: experimental and theoretical assessment of [Np(vi)O_2_Cl_4_]^2–^ and [U(vi)O_2_Cl_4_]^2–^ systems. Dalton Trans. 2019, 48, 8861–8871. 10.1039/C9DT01753D. 31139781

[ref70] Rabinowitch E.; Belford R. L. Spectroscopy and photochemistry of uranyl compounds; Pergamon: Oxford, New York, 1964.

[ref71] Schnaars D. D.; Wilson R. E. Structural and Vibrational Properties of U(VI)O_2_Cl_4_^2–^ and Pu(VI)O_2_Cl_4_^2–^ Complexes. Inorg. Chem. 2013, 52, 14138–14147. 10.1021/ic401991n. 24256199

[ref72] Schnaars D. D.; Wilson R. E. Lattice Solvent and Crystal Phase Effects on the Vibrational Spectra of UO_2_Cl_4_^2–^. Inorg. Chem. 2014, 53, 11036–11045. 10.1021/ic501553m. 25299307

[ref73] Pyrch M. M.; Williams J. M.; Kasperski M. W.; Applegate L. C.; Forbes T. Z. Synthesis and spectroscopic characterization of actinyl(VI) tetrahalide coordination compounds containing 2,2′-bipyridine. Inorg. Chim. Acta 2020, 508, 119628 10.1016/j.ica.2020.119628.

[ref74] Reeder R. J.; Nugent M.; Lamble G. M.; Tait C. D.; Morris D. E. Uranyl Incorporation into Calcite and Aragonite: XAFS and Luminescence Studies. Environ. Sci. Technol. 2000, 34, 638–644. 10.1021/es990981j.

[ref75] Ikeda A.; Hennig C.; Tsushima S.; Takao K.; Ikeda Y.; Scheinost A. C.; Bernhard G. Comparative Study of Uranyl(VI) and -(V) Carbonato Complexes in an Aqueous Solution. Inorg. Chem. 2007, 46, 4212–4219. 10.1021/ic070051y. 17417836

[ref76] Bonales L.; Colmenero F.; Cobos J.; Timón V. Spectroscopic Raman characterization of rutherfordine: a combined DFT and experimental study. Phys. Chem. Chem. Phys. 2016, 18, 16575–16584. 10.1039/C6CP01510G. 27271869

[ref77] Kalashnyk N.; Perry D. L.; Massuyeau F.; Faulques E. Exploring optical and vibrational properties of the uranium carbonate andersonite with spectroscopy and first-principles calculations. J. Phys. Chem. C 2018, 122, 7410–7420. 10.1021/acs.jpcc.8b00871.

[ref78] Suryawanshi Yogeshwar R.; Chakraborty M.; Jauhari S.; Mukhopadhyay S.; Shenoy Kalsanka T. Hydrogenation of Dibenzo-18-Crown-6 Ether Using γ-Al_2_O_3_ Supported Ru-Pd and Ru-Ni Bimetallic Nanoalloy Catalysts. Int. J. Chem. React. Eng. 2019, 17, 0049 10.1515/ijcre-2018-0049.

