# Supplementary material for: Investigations of the Cobalt Hexamine Uranyl Carbonate System: Understanding the Influence of Charge and Hydrogen Bonding on the Modification of Vibrational Modes in Uranyl Compounds
Source: Inorg Chem. 2022 Sep 13;61(38):15023–36. doi: 10.1021/acs.inorgchem.2c01982 (PMC9516682; doi:10.1021/acs.inorgchem.2c01982)
Supplement: Supplementary file 1 — ic2c01982_si_001.pdf [file ic2c01982_si_001.pdf]

Supporting Information

Investigations of the Cobalt Hexamine Uranyl Carbonate System: Understanding the Influence of Charge and Hydrogen Bonding on the Activation of Vibrational Modes in Uranyl Compounds

Mikaela M. Pyrch, Jennifer L. Bjorklund, James M. Williams, Maguire Kasperski, Sara Mason, and Tori Z. Forbes*

Department of Chemistry, University of Iowa, Iowa City, IA 52242, United States

* corresponding author; tori-forbes@uiowa.edu

Table of Contents

Crystallographic Details........3

Figure S1: Thermal ellipsoid plot of **Co_Cl_CO$_3$** at 50% probability........3

Figure S2: Thermal ellipsoid plot of **Co4U3** at 50% probability........4

Figure S3: Thermal ellipsoid plot of **Co3U2_Cl** at 50% probability. N6 and N7 are disordered at 50% site occupancy........5

Figure S4: Thermal ellipsoid plot of **Co2U1_Cl** at 50% probability........6

Figure S5: Thermal ellipsoid plot of **Co2_U1_CO$_3$** at 50% probability. The nitrogen groups associated bonded to the Co2 site are disordered with 50% occupancy. Similarly the O atom on the C1 carbonate (O5A and O5B) and the water in the lattice (OW1A, OW1B, and OW1C) are also disordered with partial occupancy refined in the structure........7

Table S1: Select bond angles (˚) and lengths (Å) for **Co_Cl_CO$_3$**........8

Table S2: Select bond angles(˚) and lengths (Å) for **Co4U3**........9

Table S3: Select bond angles (˚) and lengths (Å) for **Co3U2_Cl**........10

Table S4: Select bond angles (˚) and lengths (Å) for **Co2U1_Cl**........11

Table S5: Select bond angles (˚) and lengths (Å) for **Co2U1_CO$_3$**........12

Table S6: Select Donor-Acceptor distances of Co_Cl_CO$_3$........13

Table S7: Select Donor-Acceptor distances of Co4U3........13

Table S8: Select Donor-Acceptor distances of **Co3U2_Cl.**........13

Table S9: Select Donor-Acceptor distances of **Co2U1_Cl**........14

Table S10: Select Donor-Acceptor distances of **Co2U1_CO$_3$**........14

Vibrational Spectroscopy........15

Table S11: Fitted Raman values including peak centroid, full width half max (FWHM), and area for **Co4U3**........15

Table S12: Fitted Raman values including peak centroid, full width half max (FWHM), and area for **Co3U2Cl**.....15

Table S13: Fitted Raman values including peak centroid, full width half max (FWHM), and area for **Co2U1Cl.**....15

Table S14: Fitted Raman values including peak centroid, full width half max (FWHM), and area for **Co2U1CO3.**16

Table S15: Fitted FTIR values including peak centroid, full width half max (FWHM), and area for **Co4U3.**........16

Table S16: Fitted FTIR values including peak centroid, full width half max (FWHM), and area for **Co3U2Cl**........16

Table S17: Fitted FTIR values including peak centroid, full width half max (FWHM), and area for **Co2U1Cl**........17

Table S18: Fitted FTIR values including peak centroid, full width half max (FWHM), and area for **Co2U1CO3.**...17
Figure S6: Full FTIR Spectra of **CO_Cl_CO_3**...18
Powder X-ray diffraction...19
Figure S7: PXRD pattern for experimental (black) and the predicted (red) for **CoCl_CO_3**...19
Figure S8: PXRD pattern for experimental (black) and the predicted (red) for **Co4U3**...20
Figure S9: PXRD pattern for experimental (black) and the predicted (red) for **Co2_U1_Cl.**...21
Figure S10: PXRD pattern for experimental (black) and the predicted (red) for **Co2_U1_Cl.**...22
Computational Details...23
Figure S11: Figure of the uranyl tricarbonate computational structures used for vibrational calculations below. A) $UO_2(CO_3)_3$ short and long oxo, $Co(NH)_3$ interacting with the shorter oxo. B) $UO_2(CO_3)_3$ short and long oxo, $Co(NH)_3$ interacting with the longer oxo. C) $UO_2(CO_3)_3$ equivalent oxo lengths with no interaction. D) $UO_2(CO_3)_3$ short and long oxo, 2x $Co(NH)_3$ interacting with the longer oxo E) $UO_2(CO_3)_3$ short and long oxo, $Co(NH)_3$ interacting with the shorter oxo and the longer oxo....23
Table S19: TZVP Vibrational Analysis Results for $[UO_2(CO_3)_3]^{4-}$....24
Table S20: TZVP vibrational analysis results for $[UO_2(CO_3)_3]^{4-}$ short and long oxo, $[Co(NH)_3]^{3+}$ interacting with the shorter oxo (model depicted in Fig S11A)....24
Table S21: TZVP vibrational analysis results for $[UO_2(CO_3)_3]^{4-}$ and $[Co(NH)_3]^{3+}$ interacting with longer oxo (model depicted in Fig S11B)....25
Table S22: TZVP Vibrational analysis results for $[UO_2(CO_3)_3]^{4-}$ equivalent oxo lengths with no interaction....25
Table S23: TZVP Vibrational Analysis for $[UO_2(CO_3)_3]^{4-}$ with one short and one long oxo and two $[Co(NH)_3]^{3+}$ interacting with the longer oxo (model depicted in Fig. S11C)....26
Table S24: TZVP Vibrational Analysis for $[UO_2(CO_3)_3]^{4-}$ with one short and one long oxo and two $[Co(NH)_3]^{3+}$ interacting with the shorter oxo and the longer oxo (model depicted in Fig. S11D)....26

Crystallographic Details

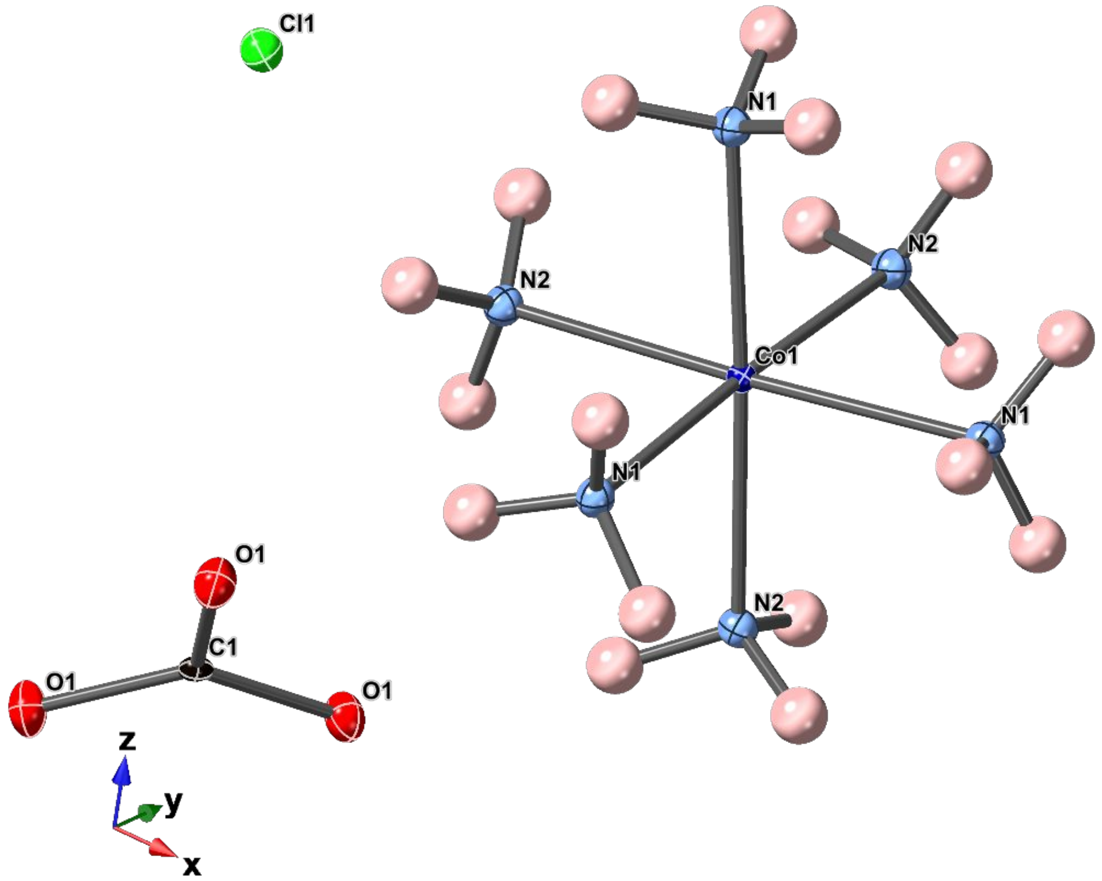

Figure S1: Thermal ellipsoid plot of **Co_Cl_CO$_3$** at 50% probability.

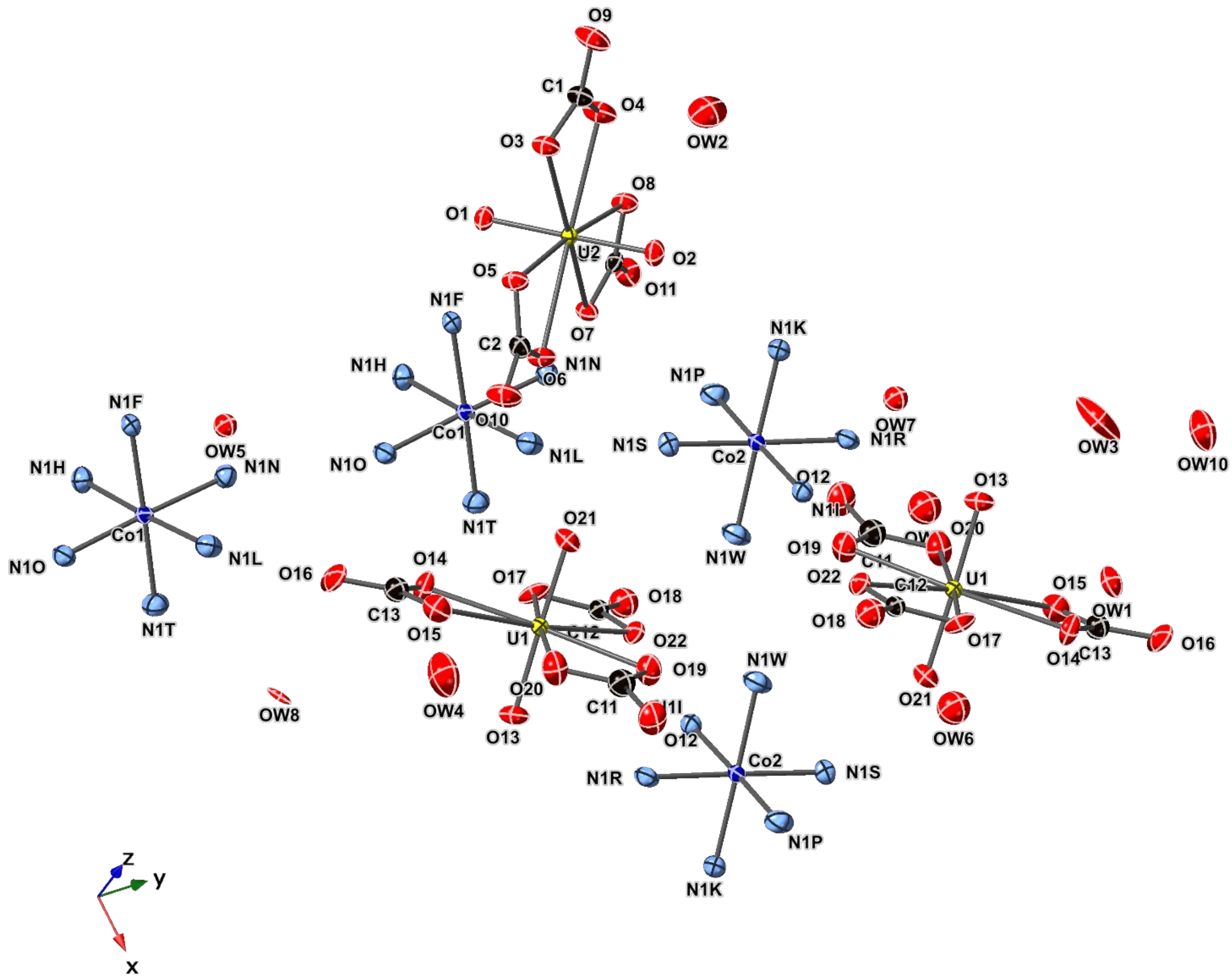

Figure S2: Thermal ellipsoid plot of **Co4U3** at 50% probability.

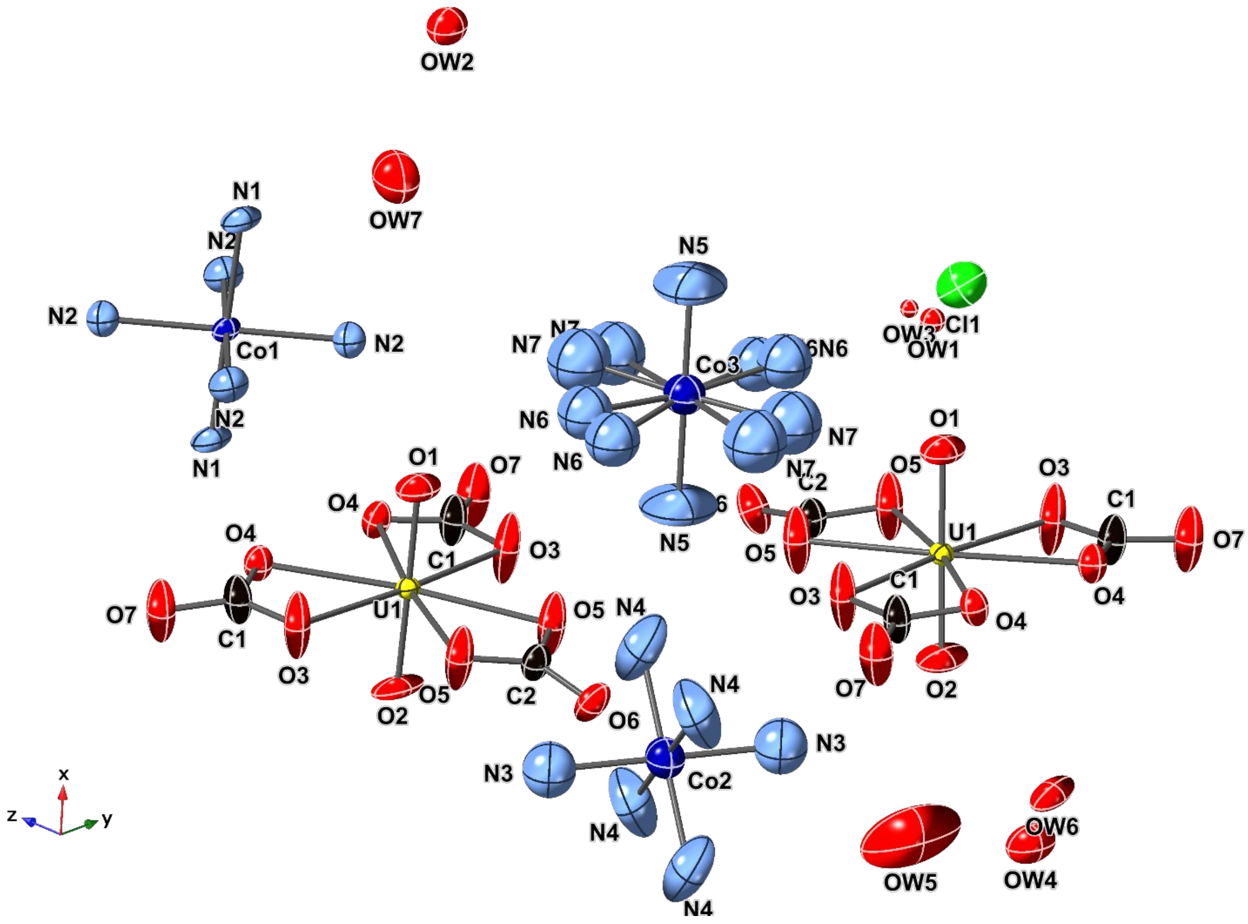

Figure S3: Thermal ellipsoid plot of **Co3U2_Cl** at 50% probability. N6 and N7 are disordered at 50% site occupancy.

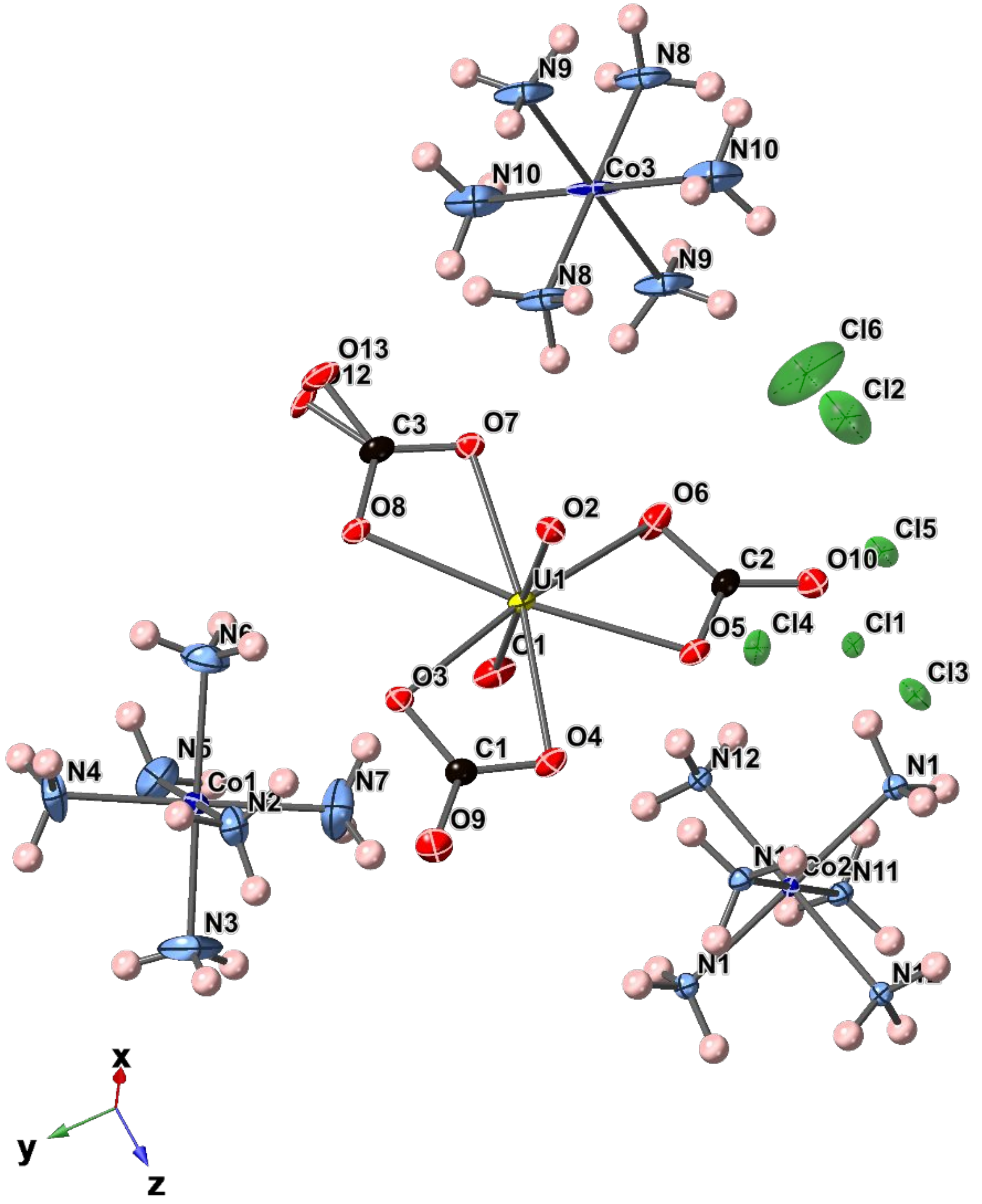

Figure S4: Thermal ellipsoid plot of **Co2U1_Cl** at 50% probability.

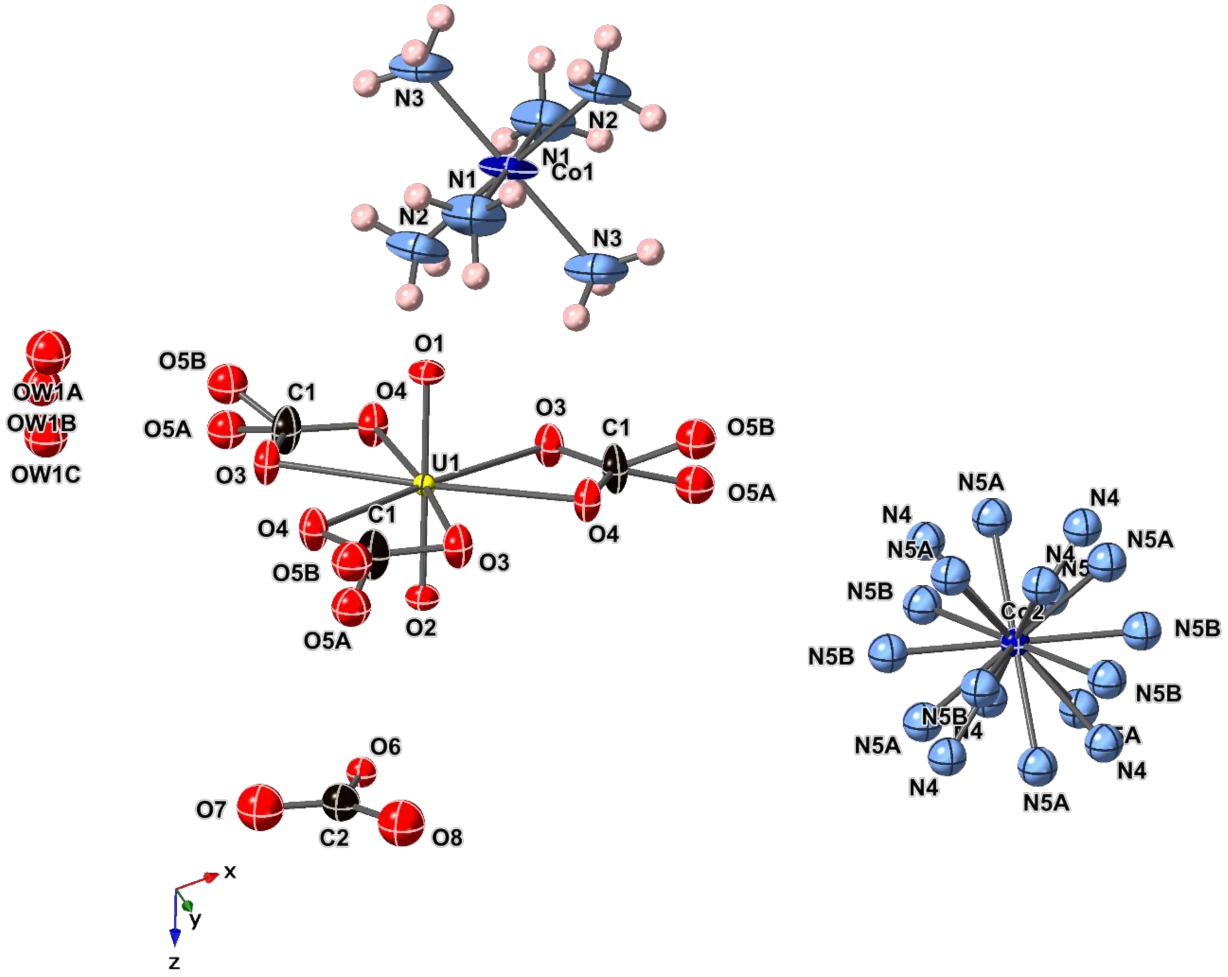

Figure S5: Thermal ellipsoid plot of **Co2_U1_CO$_3$** at 50% probability. The nitrogen groups associated bonded to the Co2 site are disordered with 50% occupancy. Similarly the O atom on the C1 carbonate (O5A and O5B) and the water in the lattice (OW1A, OW1B, and OW1C) are also disordered with partial occupancy refined in the structure.

Table S1: Select bond angles (˚) and lengths (Å) for **Co_Cl_CO_3**.

Co(1)-N(1)$^{\#1}$	1.9629(19)	N(1)$^{\#1}$-Co(1)-N(1)$^{\#2}$	91.63(9)
Co(1)-N(1)$^{\#2}$	1.9629(19)	N(1)$^{\#1}$-Co(1)-N(2)	177.41(9)
Co(1)-N(1)	1.9629(19)	N(1)$^{\#2}$-Co(1)-N(2)	87.31(9)
Co(1)-N(2)	1.9634(19)	N(1)-Co(1)-N(2)	90.76(8)
Co(1)-N(2)$^{\#2}$	1.9634(19)	N(1)-Co(1)-N(2)$^{\#2}$	177.41(9)
Co(1)-N(2)$^{\#1}$	1.9634(19)	N(2)-Co(1)-N(2)$^{\#2}$	90.33(9)
O(1)-C(1)	1.2871(18)	N(1)$^{\#1}$-Co(1)-N(2)$^{\#1}$	90.76(8)
		N(1)$^{\#2}$-Co(1)-N(2)$^{\#1}$	177.41(9)
		N(1)-Co(1)-N(2)$^{\#1}$	87.31(9)
		N(2)-Co(1)-N(2)$^{\#1}$	90.34(9)
		O(1)-C(1)-O(1)$^{\#3}$	119.999(2)
		O(1)-C(1)-O(1)$^{\#4}$	119.997(2)
		O(1)$^{\#3}$-C(1)-O(1)$^{\#4}$	119.998(2)

Symmetry transformations used to generate equivalent atoms:

$^{\#1}$ -y+1,z-1/2,-x+3/2 $^{\#2}$ -z+3/2,-x+1,y+1/2 $^{\#3}$ -z+1,x-1/2,-y+1/2 $^{\#4}$ y+1/2,-z+1/2,-x+1

Table S2: Select bond angles(˚) and lengths (Å) for **Co4U3**.

U(2)-O(2)	1.796(3)	O(2)-U(2)-O(1)	179.37(13)	N(1N)-Co(1)-N(1F)	90.53(14)
U(2)-O(1)	1.798(3)	O(2)-U(2)-O(6)	87.19(12)	N(1F)-Co(1)-N(1H)	88.49(15)
U(2)-O(6)	2.412(3)	O(1)-U(2)-O(6)	93.44(12)	N(1N)-Co(1)-N(1T)	90.36(15)
U(2)-O(7)	2.416(3)	O(2)-U(2)-O(7)	94.25(11)	N(1F)-Co(1)-N(1T)	178.65(15)
U(2)-O(4)	2.427(3)	O(1)-U(2)-O(7)	86.03(11)	N(1H)-Co(1)-N(1T)	90.49(16)
U(2)-O(5)	2.429(3)	O(1)-U(2)-O(8)	90.80(12)	N(1N)-Co(1)-N(1L)	88.20(15)
U(2)-O(3)	2.434(3)	O(2)-U(2)-O(4)	88.74(12)	N(1T)-Co(1)-N(1L)	91.21(16)
O(3)-C(1)	1.302(5)	O(1)-U(2)-O(4)	90.64(12)	N(1N)-Co(1)-N(1O)	178.34(15)
O(6)-C(2)	1.305(5)	O(8)-U(2)-O(4)	66.06(9)	N(1P)-Co(2)-N(1I)	177.63(17)
O(7)-C(3)	1.304(5)	O(2)-U(2)-O(5)	89.87(12)	N(1P)-Co(2)-N(1R)	88.00(17)
O(8)-C(3)	1.294(5)	O(1)-U(2)-O(5)	90.47(12)	N(1I)-Co(2)-N(1K)	92.04(14)
U(1)-O(12)	1.348(7)	O(7)-U(2)-O(5)	120.92(9)	N(1P)-Co(2)-N(1W)	90.16(16)
U(1)-O(21)	1.781(5)	O(4)-U(2)-O(5)	119.42(9)	N(1I)-Co(2)-N(1W)	89.21(15)
U(1)-O(13)	1.793(5)	O(2)-U(2)-O(3)	91.41(12)	N(1S)-Co(2)-N(1W)	91.84(17)
U(1)-O(22)	2.409(6)	O(1)-U(2)-O(3)	88.24(11)	N(1R)-Co(2)-N(1W)	89.85(16)
U(1)-O(17)	2.424(5)	O(6)-U(2)-O(3)	119.99(9)	O(9)-C(1)-O(4)	123.7(4)
U(1)-O(14)	2.440(6)	O(7)-U(2)-O(3)	170.75(9)	O(9)-C(1)-O(3)	122.0(4)
U(1)-O(15)	2.441(6)	O(8)-U(2)-O(3)	119.21(9)	O(4)-C(1)-O(3)	114.3(3)
O(12)-C(11)	1.235(17)	O(19)-U(1)-O(12)	145.8(5)	O(10)-C(2)-O(5)	123.0(4)
O(13)-C(11)	1.663(17)	O(19)-U(1)-O(20)	112.1(4)	O(10)-C(2)-O(6)	122.8(4)
O(15)-C(13)	1.264(11)	O(12)-U(1)-O(20)	97.9(4)	O(5)-C(2)-O(6)	114.2(3)
O(16)-C(13)	1.230(11)	O(19)-U(1)-O(21)	106.1(4)	O(11)-C(3)-O(7)	122.7(3)
O(17)-C(12)	1.310(10)	O(12)-U(1)-O(21)	87.7(4)	O(8)-C(3)-O(7)	114.3(3)
O(18)-C(12)	1.193(13)	O(20)-U(1)-O(21)	92.9(3)	O(12)-C(11)-O(20)	123.0(13)
Co(1)-N(1N)	1.959(3)	O(21)-U(1)-O(13)	178.3(3)	O(12)-C(11)-O(19)	123.1(13)
Co(1)-N(1F)	1.963(3)	O(19)-U(1)-O(22)	141.0(4)	O(20)-C(11)-O(19)	112.6(12)
Co(1)-N(1T)	1.971(4)	O(21)-U(1)-O(22)	88.9(2)	O(12)-C(11)-O(13)	104.9(11)
Co(1)-N(1O)	1.973(3)	O(13)-U(1)-O(22)	90.9(2)		
Co(2)-N(1P)	1.956(4)	O(19)-U(1)-O(17)	156.7(4)		
Co(2)-N(1I)	1.957(3)	O(21)-U(1)-O(17)	90.0(2)		
Co(2)-N(1R)	1.967(4)	O(13)-U(1)-O(17)	91.2(2)		
Co(2)-N(1K)	1.969(3)	O(20)-U(1)-O(14)	148.8(3)		

Table S3: Select bond angles (˚) and lengths (Å) for **Co3U2_Cl**.

U(1)-O(2)	1.762(11)	O(2)-U(1)-C(2)	87.3(4)
U(1)-O(1)	1.782(11)	O(1)-U(1)-C(2)	93.4(4)
U(1)-O(3)	2.413(10)	O(3)-U(1)-C(2)	93.19(18)
U(1)-O(5)	2.422(8)	O(4)-U(1)-C(2)	146.00(19)
U(1)-O(4)	2.438(7)	O(3)-C(1)-O(7)$^{\#1}$	121.8(10)
O(3)-C(1)	1.255(13)	O(3)-C(1)-O(4)	116.4(10)
O(4)-C(1)	1.286(13)	O(7)$^{\#1}$-C(1)-O(4)	121.7(9)
O(5)-C(2)	1.274(11)	O(5)-C(2)-O(6)	123.3(6)
O(6)-C(2)	1.291(17)	O(2)-U(1)-C(1)	88.6(3)
Co(1)-N(2)	1.976(8)	O(1)-U(1)-C(1)	91.0(3)
Co(1)-N(1)	1.980(10)	N(2)-Co(1)-N(1)	91.1(3)
Co(2)-N(4)	1.912(16)	N(2)$^{\#2}$-Co(1)-N(1)	88.9(3)
Co(2)-N(3)	1.97(2)	N(1)$^{\#3}$-Co(1)-N(1)	180.0
Co(3)-N(5)	1.920(18)	N(4)$^{\#6}$-Co(2)-N(4)	90.7(11)
Co(3)-N(7)	1.99(3)	N(4)$^{\#4}$-Co(2)-N(3)	89.1(7)
Co(3)-N(6)	2.00(3)	N(4)-Co(2)-N(3)	90.9(7)
O(2)-U(1)-O(1)	179.3(5)	N(5)-Co(3)-N(7)	88.8(18)
O(2)-U(1)-O(3)	89.3(3)	N(5)$^{\#7}$-Co(3)-N(7)	91.2(18)
O(1)-U(1)-O(3)	90.7(3)	N(7)$^{\#6}$-Co(3)-N(7)	178(4)
O(3)$^{\#1}$-U(1)-O(3)	173.4(4)	N(7)$^{\#8}$-Co(3)-N(7)	157.1(19)
O(2)-U(1)-O(5)	89.0(4)	N(7)$^{\#7}$-Co(3)-N(7)	23.0(19)
O(1)-U(1)-O(5)	91.6(4)	N(5)-Co(3)-N(6)	86.3(11)
O(3)$^{\#1}$-U(1)-O(5)	67.2(3)	N(5)$^{\#7}$-Co(3)-N(6)	93.7(11)
O(3)-U(1)-O(5)	119.3(3)	N(7)$^{\#6}$-Co(3)-N(6)	69.5(12)
O(5)-U(1)-O(5)$^{\#1}$	52.1(4)	N(7)$^{\#8}$-Co(3)-N(6)	92.5(13)
O(1)-U(1)-O(4)$^{\#1}$	89.5(3)	N(7)$^{\#7}$-Co(3)-N(6)	87.6(13)
O(3)-U(1)-O(4)$^{\#1}$	120.7(3)	N(7)-Co(3)-N(6)	110.3(12)
O(2)-U(1)-O(4)	89.9(3)		
O(3)$^{\#1}$-U(1)-O(4)	120.7(3)		
O(3)-U(1)-O(4)	52.9(3)		
O(5)-U(1)-O(4)	172.1(3)		

Symmetry transformations used to generate equivalent atoms: $^{\#1}$ x,-y+1/2,z $^{\#2}$ -x+1/2,y+0,-z $^{\#3}$ -x+1/2,-y+1/2,-z $^{\#4}$ -x+1,-y+1,-z+1 $^{\#5}$ -x+1,y,z $^{\#6}$ x,-y+1,-z+1 $^{\#7}$ -x+1/2,-y+1,z $^{\#8}$ -x+1/2,y+0,-z+1 $^{\#9}$-x+1,-y+1/2,z

Table S4: Select bond angles (˚) and lengths (Å) for **Co2U1_Cl**.

U(1)-O(1)	1.798(4)	O(1)-U(1)-O(2)	179.03(19)	N(8)-Co(1)-N(9)	90.9(2)
U(1)-O(2)	1.803(4)	O(1)-U(1)-O(8)	91.61(18)	N(10)-Co(1)-N(9)	88.0(3)
U(1)-O(8)	2.394(4)	O(2)-U(1)-O(8)	89.14(16)	N(4)-Co(2)-N(7)	178.3(3)
U(1)-O(5)	2.403(4)	O(1)-U(1)-O(5)	92.01(18)	N(4)-Co(2)-N(6)	88.1(3)
U(1)-O(6)	2.428(4)	O(2)-U(1)-O(5)	87.18(16)	N(7)-Co(2)-N(6)	91.5(3)
U(1)-O(3)	2.438(4)	O(8)-U(1)-O(5)	173.00(14)	N(4)-Co(2)-N(3)	93.2(4)
U(1)-O(4)	2.439(4)	O(1)-U(1)-O(6)	86.8(2)	N(7)-Co(2)-N(3)	87.3(4)
U(1)-C(3)	2.863(6)	O(2)-U(1)-O(6)	92.32(17)	N(6)-Co(2)-N(3)	178.6(3)
U(1)-C(2)	2.871(6)	O(8)-U(1)-O(6)	120.22(14)	N(4)-Co(2)-N(5)	87.9(2)
U(1)-C(1)	2.886(6)	O(5)-U(1)-O(6)	54.05(14)	N(7)-Co(2)-N(5)	90.4(3)
Co(1)-N(8)	1.947(5)	O(1)-U(1)-O(3)	91.22(18)	N(6)-Co(2)-N(5)	91.1(3)
Co(1)-N(10)	1.963(7)	O(2)-U(1)-O(3)	89.65(16)	N(3)-Co(2)-N(5)	89.6(3)
Co(1)-N(9)	1.976(6)	O(8)-U(1)-O(3)	65.85(13)	N(4)-Co(2)-N(2)	91.5(2)
Co(2)-N(4)	1.936(6)	O(5)-U(1)-O(3)	120.05(13)	N(7)-Co(2)-N(2)	90.1(2)
Co(2)-N(7)	1.946(6)	O(6)-U(1)-O(3)	173.63(14)	N(6)-Co(2)-N(2)	89.0(3)
Co(2)-N(6)	1.948(6)	O(1)-U(1)-O(7)	91.04(17)	N(3)-Co(2)-N(2)	90.4(3)
Co(2)-N(3)	1.953(6)	O(2)-U(1)-O(7)	88.91(16)	N(5)-Co(2)-N(2)	179.4(2)
Co(2)-N(5)	1.959(5)	O(8)-U(1)-O(7)	53.72(14)	N(12)-Co(3)-N(11)	88.3(2)
Co(2)-N(2)	1.963(5)	O(5)-U(1)-O(7)	120.20(14)	N(12)-Co(3)-N(1)	90.54(19)
Co(3)-N(12)	1.955(4)	O(6)-U(1)-O(7)	66.56(14)	N(11)-Co(3)-N(1)	90.6(2)
Co(3)-N(11)	1.960(5)	O(3)-U(1)-O(7)	119.57(13)	O(9)-C(1)-O(4)	123.4(5)
Co(3)-N(1)	1.970(5)	O(1)-U(1)-O(4)	87.33(17)	O(9)-C(1)-O(3)	122.3(6)
O(3)-C(1)	1.310(7)	O(2)-U(1)-O(4)	92.84(16)	O(4)-C(1)-O(3)	114.2(5)
O(4)-C(1)	1.298(8)	O(8)-U(1)-O(4)	119.14(14)	O(10)-C(2)-O(6)	124.8(6)
O(5)-C(2)	1.320(7)	O(5)-U(1)-O(4)	67.04(14)	O(10)-C(2)-O(5)	121.7(5)
O(6)-C(2)	1.305(7)	O(6)-U(1)-O(4)	120.45(14)	O(6)-C(2)-O(5)	113.5(5)
O(9)-C(1)	1.252(8)	O(7)-U(1)-O(4)	172.66(14)	O(11)-C(3)-O(7)	123.0(6)
O(10)-C(2)	1.234(7)			O(11)-C(3)-O(8)	122.8(6)
O(11)-C(3)	1.225(8)				

Table S5: Select bond angles (˚) and lengths (Å) for **Co2U1_CO$_3$**.

U(1)-O(1)	1.771(8)	O(1)-U(1)-O(2)	180.0
U(1)-O(2)	1.776(8)	O(1)-U(1)-O(3)	89.96(13)
U(1)-O(3)$^{\#1}$	2.435(5)	O(2)-U(1)-O(3)	90.04(13)
U(1)-O(3)	2.435(5)	O(3)-U(1)-O(4)$^{\#1}$	66.70(16)
U(1)-O(4)	2.439(5)	O(3)-U(1)-O(4)$^{\#2}$	173.30(16)
U(1)-C(1)$^{\#1}$	2.881(7)	O(1)-U(1)-O(4)	90.01(13)
U(1)-C(1)	2.881(7)	O(2)-U(1)-O(4)	89.99(13)
O(3)-C(1)	1.304(9)	O(3)$^{\#1}$-U(1)-O(4)	173.30(16)
O(4)-C(1)	1.296(9)	O(3)-U(1)-O(4)	53.30(16)
O(5A)-C(1)	1.273(14)	O(5A)-C(1)-O(4)	121.5(8)
O(5B)-C(1)	1.277(14)	O(5B)-C(1)-O(4)	121.5(8)
Co(1)-N(1)	1.939(6)	O(5A)-C(1)-O(3)	120.9(8)
Co(1)-N(2)	1.970(8)	O(5B)-C(1)-O(3)	121.1(8)
Co(1)-N(3)	1.970(8)	O(4)-C(1)-O(3)	114.5(6)
Co(2)-N(5B)$^{\#4}$	1.92(2)	N(1)-Co(1)-N(2)	90.0(3)
Co(2)-N(5B)	1.92(2)	N(1)$^{\#3}$-Co(1)-N(3)	89.9(3)
Co(2)-N(5A)	1.97(2)	N(1)-Co(1)-N(3)	90.1(3)
C(2)-O(7)	1.213(16)	N(2)-Co(1)-N(3)	92.7(4)
C(2)-O(6)	1.213(9)	N(2)$^{\#3}$-Co(1)-N(3)	87.3(4)
C(2)-O(8)	1.214(16)	N(5B)$^{\#5}$-Co(2)-N(5A)	126.4(9)
		N(5B)-Co(2)-N(5A)	52.6(9)
O(7)-C(2)-O(6)	117.8(17)	N(5B)$^{\#4}$-Co(2)-N(5A)$^{\#5}$	127.4(9)
O(7)-C(2)-O(8)	123.6(17)	N(5B)$^{\#5}$-Co(2)-N(5A)$^{\#5}$	52.6(9)
O(6)-C(2)-O(8)	118.6(17)	N(5A)-Co(2)-N(5A)$^{\#5}$	73.8(9)
		N(5B)$^{\#4}$-Co(2)-N(5A)$^{\#7}$	90.2(9)
		N(5B)$^{\#5}$-Co(2)-N(5A)$^{\#7}$	89.8(9)
		N(5B)$^{\#6}$-Co(2)-N(5A)$^{\#7}$	126.4(9)
		N(5B)$^{\#8}$-Co(2)-N(5A)$^{\#7}$	127.4(9)
		N(5A)-Co(2)-N(5A)$^{\#7}$	106.2(9)

Symmetry transformations used to generate equivalent atoms: $^{\#1}$ -x+y,-x+1,z $^{\#2}$ -y+1,x-y+1,z $^{\#3}$ -x+1,-y+2,-z $^{\#4}$ x-y,x,-z $^{\#5}$ -x+y,-x,z $^{\#6}$ -x,-y,-z $^{\#7}$ y,-x+y,-z $^{\#8}$ -y,x-y,z

Table S6: Select Donor-Acceptor distances of Co_Cl_CO_3

Donor	Acceptor	Distance (Å)
N1	O1	2.882
N1	O1	3.001
N2	O1	2.849

Table S7: Select Donor-Acceptor distances of Co4U3.

Donor	Acceptor	Distance (Å)	Donor	Acceptor	Distance (Å)
N1N	O4	2.965	N1L	O16	2.969
N1H	O8	3.045	N1N	O16	2.913
N1N	O8	3.014	N1S	O21	3.018
N1O	O1	2.978	N1W	O13	3.024
N1F	O1	2.957	N1P	OW8	2.657
N1L	O7	2.97	N1I	O18	2.875
N1F	O7	3.045	N1R	O22	2.868
N1N	O11	2.911	N1W	O22	2.69
N1K	O2	3.007	N1I	OW6	3.049
N1S	O6	2.958	OW3	OW8	3.014
N1P	O7	2.916	OW4	OW1	2.708
N1K	O10	2.912	OW5	O14	2.955
N1P	O10	2.871	OW5	O16	2.891
N1R	O10	2.938	OW5	OW1	1.94
N1I	O3	2.918	OW7	OW1	2.643
N1K	O3	3.066	OW3	N1O	2.947
OW2	O9	2.778	OW7	N1T	2.839
OW3	O11	2.742	OW4	N1I	3.05
OW3	O6	2.809	OW10	N1S	2.926
OW4	O9	2.734	OW5	OW2	2.868
OW10	O5	2.794	OW7	OW2	2.863

Table S8: Select Donor-Acceptor distances of **Co3U2_Cl.**

Donor	Acceptor	Distance (Å)	Donor	Acceptor	Distance (Å)
N1	O4	2.937	Cl1	O6	2.667
N2	O4	2.949	OW1	O7	2.813
N3	O2	2.930	OW3	O7	2.774
N4	O3	2.989	Cl1	O7	2.628
N4	O5	3.013	OW2	O6	2.891
N6	O1	2.956	OW7	O7	2.715
N5	O5	3.000			
N5	O3	3.040			
OW1	O6	2.906			
OW3	O6	2.759			

Table S9: Select Donor-Acceptor distances of **Co2U1_Cl**

**Donor**	**Acceptor**	**Distance (Å)**	**Donor**	**Acceptor**	**Distance (Å)**
N2	O9	2.958	OW1	O9	2.756
N3	O9	3.002	Cl2	O12	2.95
N4	O12	2.679	O13	O7	2.227
N5	O12	3.056	O13	O8	2.227
N7	O2	3.02	O13	N4	2.895
N5	O8	2.984	O13	N5	2.933
N7	O1	2.893	O13	Cl2	3.222
N2	O3	3.024	O13	Cl6	2.724
N6	O8	3.02			
N4	O10	2.773			
N12	O2	2.949			
N11	O5	3.054			
N1	O10	2.937			
N8	O2	3.066			
N8	O1	2.964			
N10	O6	2.991			

Table S10: Select Donor-Acceptor distances of **Co2U1_CO_3**

**Donor**	**Acceptor**	**Distance (Å)**	**Donor**	**Acceptor**	**Distance (Å)**
N1	O1	2.967			
N1	O2	2.968			

Vibrational Spectroscopy

Table S11: Fitted Raman values including peak centroid, full width half max (FWHM), and area for **Co4U3**.

**Peak centroid** (cm^{-1})	**FWHM** (cm^{-1})	**Area** (counts/cm^{-1})
684	8.5	1.1
719	8.6	8.5
727	5.9	5.2
805	7.2	67.6
1041	6.2	0.7
1053	7.1	12.2
1058	5.8	4.6

Table S12: Fitted Raman values including peak centroid, full width half max (FWHM), and area for **Co3U2Cl**.

**Peak centroid** (cm^{-1})	**FWHM** (cm^{-1})	**Area** (counts/cm^{-1})
734	16	13.0
753	6.8	8.9
809	8.4	55
857	8.2	0.7
896	6.9	2.7
951	13	8.3
1066	5.6	5.6
1070	5.7	3.8

Table S13: Fitted Raman values including peak centroid, full width half max (FWHM), and area for **Co2U1Cl.**

**Peak centroid** (cm^{-1})	**FWHM** (cm^{-1})	**Area** (counts/cm^{-1})
720	8.5	1.1
728	8.6	8.5
807	5.9	5.2
805	7.15	67.6
1041	8.3	3.2
1051	6.2	0.7
1058	7.1	12.2

Table S14: Fitted Raman values including peak centroid, full width half max (FWHM), and area for **Co2U1CO3.**

**Peak centroid** (cm^{-1})	**FWHM** (cm^{-1})	**Area** (counts/cm^{-1})
693	4.2	0.6
720	4.8	2.5
726	6.1	8.12
807	6.3	77.9
881	11.7	2.11
1056	7.9	8.7

Table S65: Fitted FTIR values including peak centroid, full width half max (FWHM), and area for **Co4U3.**

**Peak centroid** (cm^{-1})	**FWHM** (cm^{-1})	**Area** (counts/cm^{-1})
845	16.2	1.9
854	20.0	3.6
868	9.9	14.3
888	21.1	24.8
922	30.8	45.0
940	19.1	10.3

Table S76: Fitted FTIR values including peak centroid, full width half max (FWHM), and area for **Co3U2Cl**.

**Peak centroid** (cm^{-1})	**FWHM** (cm^{-1})	**Area** (counts/cm^{-1})
666	7.2	5.7
692	17.4	35.6
704	10.2	19.9
721	11.2	40.8
833	7.1	12.3
877	17.9	33.7
890	15.8	19.3
949	7.2	27.5
957	3.5	6.9
1009	10.7	48.2
1049	13.7	51.7

Table S8: Fitted FTIR values including peak centroid, full width half max (FWHM), and area for **Co2U1Cl**.

**Peak centroid** (cm^{-1})	**FWHM** (cm^{-1})	**Area** (counts/cm^{-1})
686	8.2	2.0
697	23.2	4.9
720	8.5	8.8
833	47.9	14.7
871	36.4	62.1
1044	14.5	7.3

Table S9: Fitted FTIR values including peak centroid, full width half max (FWHM), and area for **Co2U1CO3.**

**Peak centroid** (cm^{-1})	**FWHM** (cm^{-1})	**Area** (counts/cm^{-1})
668	3.0	8.4
691	13.1	47.1
722	6.6	44.5
827	21.4	5.3
859	36.5	28.7
888	13.4	65.9
1043	11.5	26.8

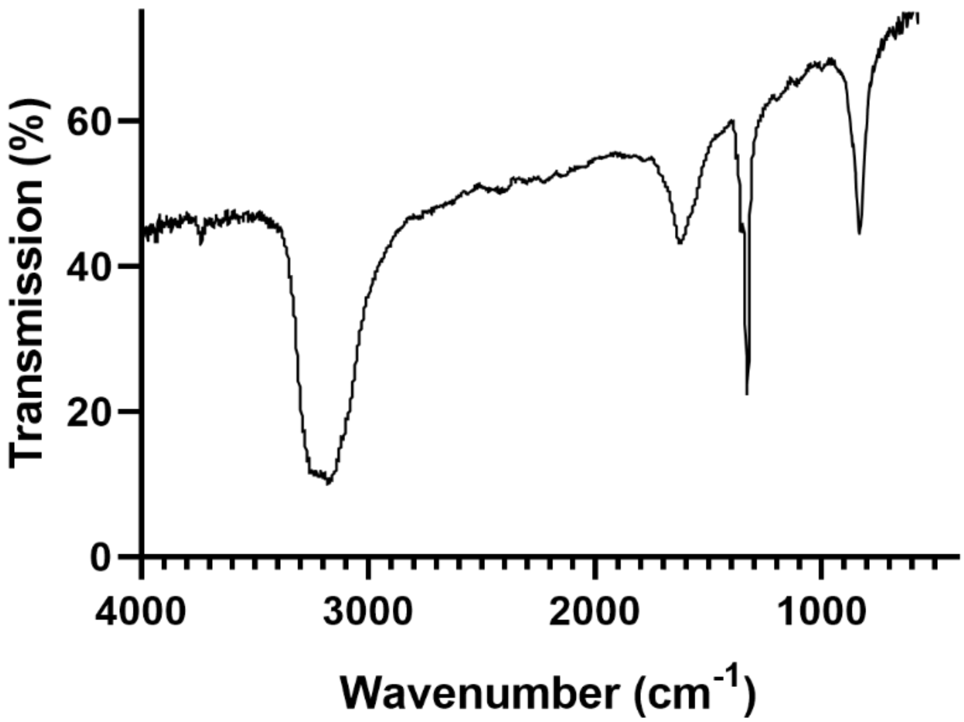

Figure S6: Full FTIR Spectra of **CO_Cl_CO_3**

Powder X-ray diffraction

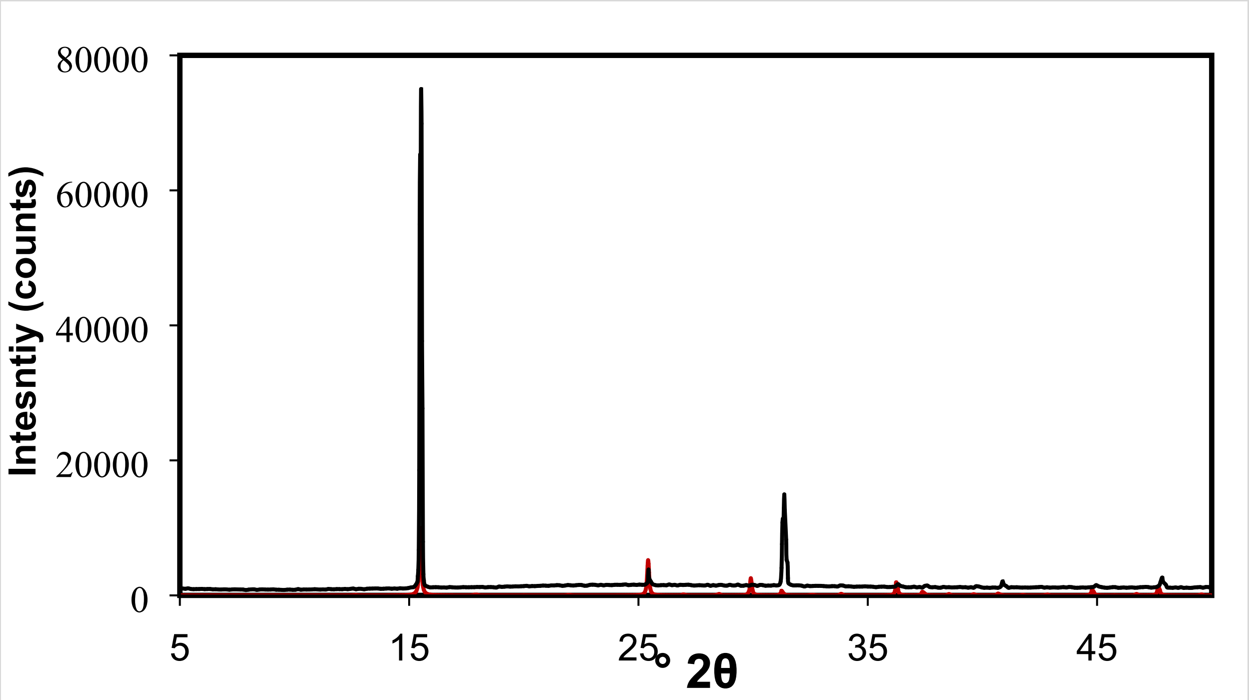

Figure S7: PXRD pattern for experimental (black) and the predicted (red) for **CoCl_CO_3**

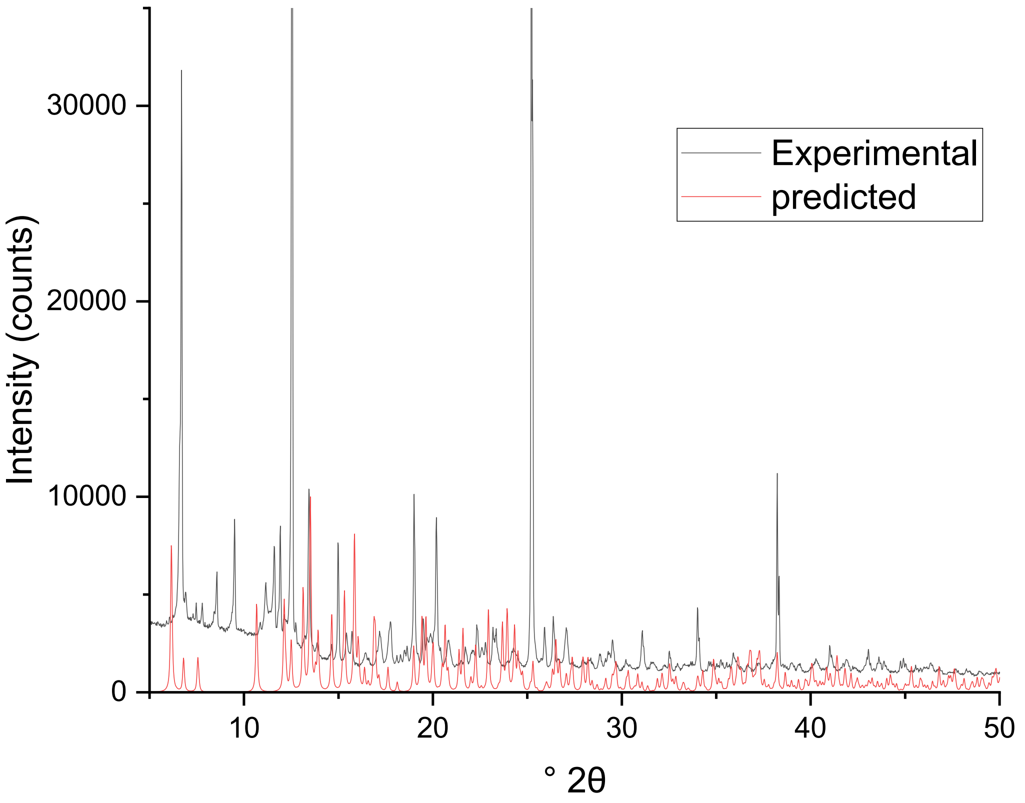

Figure S8: PXRD pattern for experimental (black) and the predicted (red) for **Co4U3**

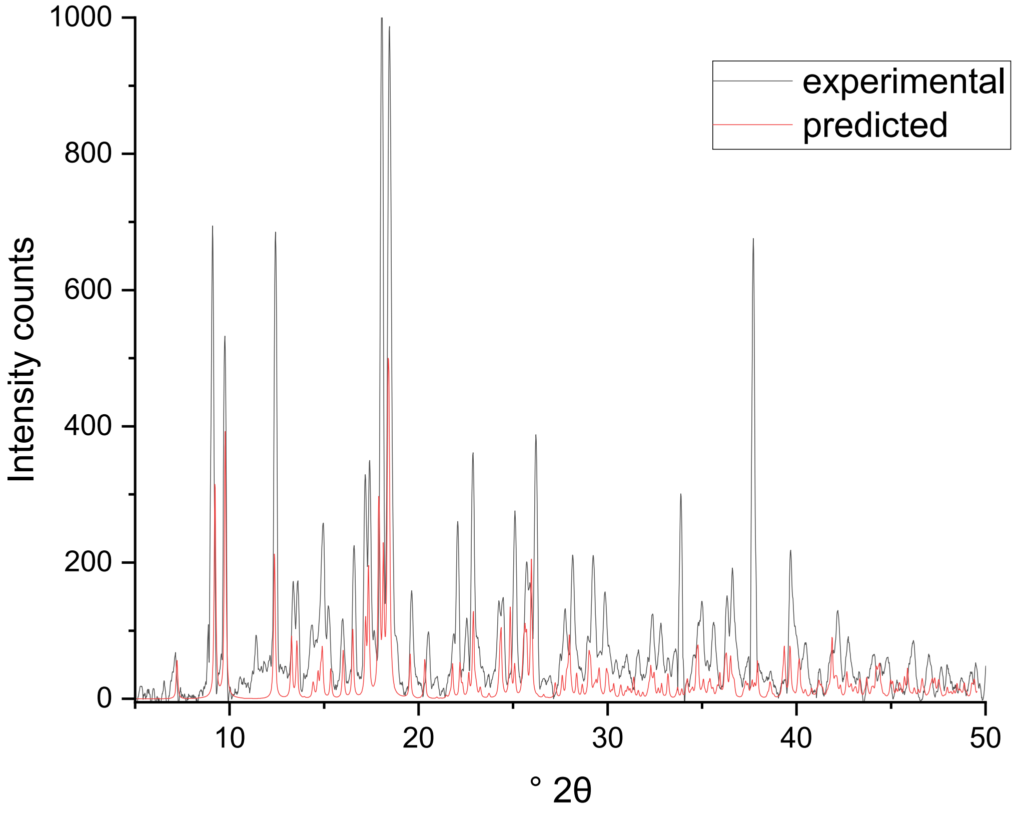

Figure S9: PXRD pattern for experimental (black) and the predicted (red) for **Co2U1_Cl.**

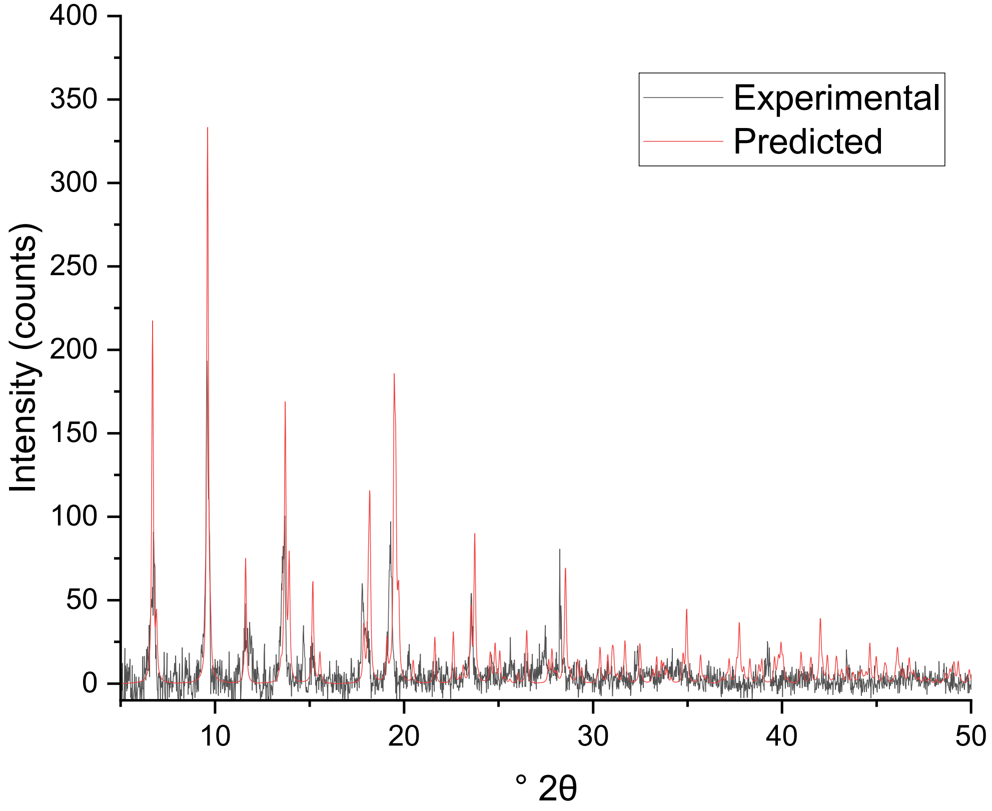

Figure S10: PXRD pattern for experimental (black) and the predicted (red) for **Co2U1_CO_3.**

Computational Details

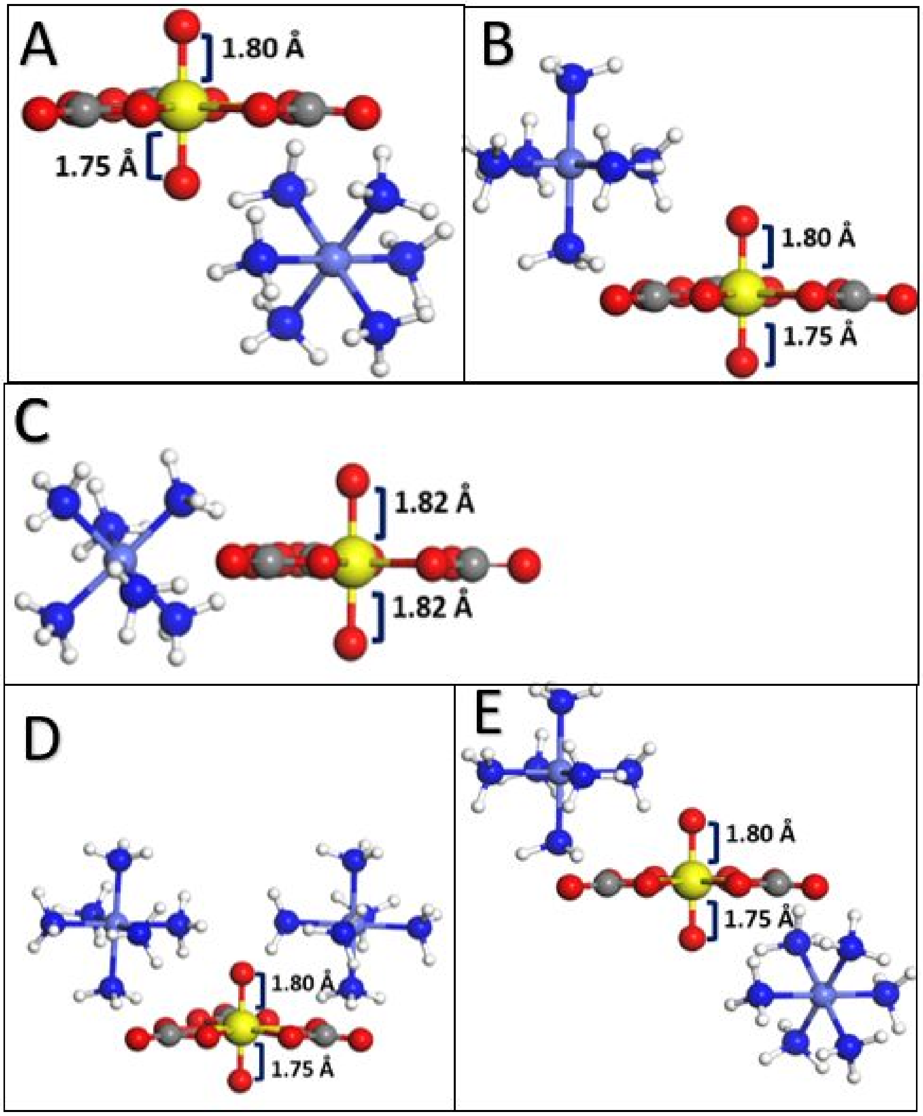

Figure S11: Figure of the uranyl tricarbonate computational structures used for vibrational calculations below. A) $UO_2(CO_3)_3$ short and long oxo, $Co(NH)_3$ interacting with the shorter oxo. B) $UO_2(CO_3)_3$ short and long oxo, $Co(NH)_3$ interacting with the longer oxo. C) $UO_2(CO_3)_3$ equivalent oxo lengths with no interaction. D) $UO_2(CO_3)_3$ short and long oxo, 2x $Co(NH)_3$ interacting with the longer oxo E) $UO_2(CO_3)_3$ short and long oxo, $Co(NH)_3$ interacting with the shorter oxo and the longer oxo.

Table S10: TZVP Vibrational Analysis Results for $[UO_2(CO_3)_3]^{4-}$.

Mode	Frequency (cm^{-1})	IR Intensity (km/mol)
**7**	670	0
**8**	679	24
**9**	680	23
**10**	715	145
**11**	715	144
**12**	717	1
**13**	789	0
**14**	830	370
**15**	835	0
**16**	836	0
**17**	839	695
**18**	1057	47
**19**	1065	0

Table S20: TZVP vibrational analysis results for $[UO_2(CO_3)_3]^{4-}$ short and long oxo, $[Co(NH)_3]^{3+}$ interacting with the shorter oxo (model depicted in Fig S11A).

Mode	Frequency (cm^{-1})	IR Intensity (km/mol)	Mode	Frequency (cm^{-1})	IR Intensity (km/mol)	Mode	Frequency (cm^{-1})	IR Intensity (km/mol)
**7**	682	0	**20**	863	2	**33**	1092	46
**8**	694	38	**21**	876	2	**34**	1095	9
**9**	724	154	**22**	960	766	**35**	1099	52
**10**	725	146	**23**	1022	31	**36**	1110	42
**11**	738	6	**24**	1027	21	**37**	1114	5
**12**	825	5	**25**	1029	1			
**13**	832	22	**26**	1056	16			
**14**	833	21	**27**	1061	9			
**15**	837	28	**28**	1072	34			
**16**	840	5	**29**	1082	147			
**17**	846	3	**30**	1084	22			
**18**	852	0	**31**	1085	30			
**19**	861	266	**32**	1090	124			

Table S21: TZVP vibrational analysis results for $[UO_2(CO_3)_3]^{4-}$ and $[Co(NH)_3]^{3+}$ interacting with longer oxo (model depicted in Fig S11B).

Mode	Frequency (cm^{-1})	IR Intensity (km/mol)	Mode	Frequency (cm^{-1})	IR Intensity (km/mol)	Mode	Frequency (cm^{-1})	IR Intensity (km/mol)
**1**	670	5	**14**	945	16	**27**	1073	34
**2**	677	63	**15**	953	11	**28**	1079	62
**3**	716	63	**16**	956	54	**29**	1087	5
**4**	799	10	**17**	984	317			
**5**	828	18	**18**	985	268			
**6**	830	6	**19**	986	323			
**7**	833	17	**20**	1008	187			
**8**	840	21	**21**	1026	54			
**9**	843	37	**22**	1027	133			
**10**	846	29	**23**	1037	255			
**11**	849	8	**24**	1043	150			
**12**	868	138	**25**	1051	131			
**13**	881	0	**26**	1072	9			

Table S22: TZVP Vibrational analysis results for $[UO_2(CO_3)_3]^{4-}$ equivalent oxo lengths with no interaction.

Mode	Frequency (cm^{-1})	IR Intensity (km/mol)	Mode	Frequency (cm^{-1})	IR Intensity (km/mol)
**1**	659	0	**14**	803	1
**2**	671	1	**15**	831	46
**3**	678	4	**16**	835	5
**4**	680	23	**17**	836	7
**5**	683	33	**18**	838	19
**6**	716	186	**19**	851	1016
**7**	718	85	**20**	855	110
**8**	724	45	**21**	888	32
**9**	736	8	**22**	908	179
**10**	739	22	**23**	1052	147
**11**	763	67	**24**	1055	13
**12**	772	52	**25**	1062	33
**13**	801	18			

Table S23: TZVP Vibrational Analysis for $[UO_2(CO_3)_3]^{4-}$ with one short and one long oxo and two $[Co(NH)_3]^{3+}$ interacting with the longer oxo (model depicted in Fig. S11C).

**Mode**	Frequency (cm^{-1})	IR Intensity (km/mol)	**Mode**	Frequency (cm^{-1})	IR Intensity (km/mol)	**Mode**	Frequency (cm^{-1})	IR Intensity (km/mol)
**1**	688	0	**14**	846	23	**27**	1036	11
**2**	698	33	**15**	849	42	**28**	1037	17
**3**	699	48	**16**	851	7	**29**	1063	1
**4**	725	163	**17**	854	46	**30**	1067	6
**5**	738	3	**18**	860	0	**31**	1068	22
**6**	805	19	**19**	863	17	**32**	1076	31
**7**	813	33	**20**	867	0	**33**	1083	19
**8**	830	4	**21**	871	105	**34**	1089	38
**9**	834	15	**22**	960	821	**35**	1090	2
**10**	835	1	**23**	1025	0	**36**	1091	96
**11**	836	5	**24**	1029	2	**37**	1092	114
**12**	843	30	**25**	1033	6	**38**	1096	111
**13**	844	54	**26**	1035	4	**39**	1099	93

Table S24: TZVP Vibrational Analysis for $[UO_2(CO_3)_3]^{4-}$ with one short and one long oxo and two $[Co(NH)_3]^{3+}$ interacting with the shorter oxo and the longer oxo (model depicted in Fig. S11D).

**Mode**	Frequency (cm^{-1})	IR Intensity (km/mol)	**Mode**	Frequency (cm^{-1})	IR Intensity (km/mol)	**Mode**	Frequency (cm^{-1})	IR Intensity (km/mol)
**1**	684	0	**14**	849	58	**27**	1031	4
**2**	695	1	**15**	853	6	**28**	1056	15
**3**	696	4	**16**	857	6	**29**	1062	5
**4**	723	19	**17**	859	2	**30**	1064	5
**5**	724	29	**18**	863	0	**31**	1071	13
**6**	736	3	**19**	864	90	**32**	1072	40
**7**	828	6	**20**	868	8	**33**	1079	12
**8**	834	11	**21**	875	4	**34**	1083	194
**9**	834	53	**22**	876	3	**35**	1084	32
**10**	840	21	**23**	961	220	**36**	1085	314
**11**	842	21	**24**	1022	8	**37**	1086	402
**12**	845	4	**25**	1028	7	**38**	1090	160
**13**	846	4	**26**	1029	2	**39**	1092	148
